# Assessment of the efficacy of Chinese herbal medicine combined with western medicine for treating severe acute pancreatitis-related acute lung injury/acute respiratory distress syndrome: a systematic review and meta-analysis based on randomized controlled trials

**DOI:** 10.3389/fphar.2025.1551652

**Published:** 2025-10-30

**Authors:** Xin Han, Ming Zhang, Congcong Dou, Lianfeng Shan, Qingqiang Ni

**Affiliations:** ^1^ Shandong University of Traditional Chinese Medicine, Jinan, Shandong, China; ^2^ Department of Hepatobiliary Surgery, Shandong Provincial Hospital Affiliated to Shandong First Medical University, Jinan, Shandong, China; ^3^ Shandong Xinzhonglu Traditional Chinese Medicine Hospital, Jinan, Shandong, China

**Keywords:** Chinese herbal medicine, severe acute pancreatitis, acute lung injury, acute respiratory distress syndrome, randomized controlled trials

## Abstract

**Purpose:**

This systematic review and meta-analysis aimed to evaluate the efficacy of Chinese herbal medicine (CHM) combined with Western medicine (WM) for treating severe acute pancreatitis-related acute lung injury/acute respiratory distress syndrome (SAP-ALI/ARDS).

**Methods:**

A comprehensive search of 12 English and Chinese databases yielded 13 randomized controlled trials (RCTs) involving 917 patients. The experimental group received CHM compounds combined with standard WM, while the control group received only WM. Outcomes included clinical efficacy, inflammatory markers (e.g., TNF-α, IL-6), disease progression indicators (e.g., time to abdominal pain relief, ICU stay), and mortality rates. The Cochrane Risk of Bias Tool was used to assess study quality, and meta-analysis was conducted using RevMan 5.4.

**Results:**

The meta-analysis demonstrated that CHM combined with WM significantly improved clinical efficacy (RR = 1.26, 95% CI: 1.17–1.37, P < 0.00001), reduced inflammatory markers (e.g., TNF-α: MD = −18.18 pg/mL, P < 0.00001; IL-6: MD = −24.70 pg/mL, P < 0.00001), and shortened disease progression indicators (e.g., time to abdominal pain relief: MD = −1.56 days, P < 0.00001; ICU stay: MD = −3.27 days, P < 0.00001). However, no significant difference in mortality rates was observed (RR = 0.47, P = 0.96).

**Conclusion:**

This study provides robust evidence that the combination of Chinese herbal medicine with Western medicine significantly enhances clinical outcomes for patients with SAP-ALI/ARDS. The findings highlight improvements in inflammatory markers, disease progression indicators, and oxygenation indices. However, the lack of significant differences in mortality rates and the limited methodological rigor of included studies (e.g., blinding and allocation concealment) are notable limitations. Future research should focus on optimizing RCT designs, exploring molecular mechanisms, and investigating long-term outcomes to strengthen the evidence base for integrated therapies.

**Systematic Review Registration:**

https://www.crd.york.ac.uk/PROSPERO/, identifer CRD42024579735.

## Introduction

Severe acute pancreatitis (SAP) is a life-threatening disease marked by rapid progression, high mortality, and frequent systemic complications (Zerem, 2014). Its development is primarily driven by a massive release of pancreatic inflammatory mediators (Guo et al., 2016), which triggers systemic inflammatory response syndrome (SIRS) and subsequent multi-organ dysfunction (Garg and Singh, 2019; Hu et al., 2023). Among extrapancreatic organs, the lungs are the most frequently affected, with 15%–60% of SAP patients developing acute lung injury (ALI) or acute respiratory distress syndrome (ARDS). The mortality rate for these patients exceeds 60% within the first week (Feng, 2017). The pathogenesis of SAP-ALI/SAP-ARDS involves multiple pathways, including pancreatic elastase-mediated pulmonary damage (Chen et al., 2024), the specific effects of pancreatic enzymes and serum phospholipase A2 (Elder et al., 2012), and the overproduction of oxidative stress factors, macrophage migration inhibitory factor, and cytokines such as Interleukin-6 (IL-6), and Tumor Necrosis Factor-α (TNF-α) (Akbarshahi et al., 2012). Additionally, gastrointestinal dysfunction plays a pivotal role in the pathological process of SAP-induced ALI/ARDS (Lijian et al., 2023). Inflammatory mediators from the pancreas can disrupt the intestinal mucosal barrier, leading to the systemic dissemination of gut-derived endotoxins and bacteria, which exacerbate pulmonary damage and contribute to multi-organ failure (Liang et al., 2021).

Clinically, SAP-ALI/ARDS patients often present with acute respiratory failure, characterized by dyspnea, cyanosis, and hypoxemia. Pathologically, these conditions are marked by reduced lung compliance, increased pulmonary vascular permeability, interstitial fibrosis, and edema. Current therapeutic strategies include oxygen therapy, mechanical ventilation, thoracocentesis, and continuous blood purification (Guangxu et al., 2022). Continuous blood purification has been shown to effectively remove inflammatory mediators and mitigate organ damage (Chen Feiyang and Xiang, 2024). However, refractory hypoxemia often requires mechanical ventilation, which may lead to complications such as ventilator-associated pneumonia, accounting for 36%–90% of ALI/ARDS-related deaths (Zhang et al., 2023).

Based on the traditional Chinese medical theory of “the lungs and large intestine being anatomically and functionally connected,” integrated Chinese and Western medicine (WM) approaches have garnered significant attention (Yunfeng et al., 2024). A prior systematic review of 18 randomized controlled trials (RCTs) found that adjunctive Chinese herbal medicine (CHM), while not reducing mortality, improved oxygenation indices and shortened mechanical ventilation duration (Ji-Ping et al., 2013). This study aims to systematically evaluate the clinical efficacy of combining CHM with conventional WM for the treatment of SAP-ALI/ARDS through quality assessment and meta-analysis, providing evidence-based recommendations for clinical practice.

## Methods

This study adheres strictly to the PRISMA (Preferred Reporting Items for Systematic Reviews and Meta-Analyses) guidelines and is registered on the PROSPERO platform under the registration number CRD42024579735.

### Search strategy

A systematic search was conducted across 12 databases, including PubMed, Cochrane Library, Web of Science, Embase, ProQuest, Scopus, OVID, Chinese Biological Medical Database (CBM), China National Knowledge Infrastructure (CNKI), Duxiu, WanFang, and VIP, to identify clinical studies on the treatment of SAP-ALI/ARDS with CHM, published up to 31 July 2024. Additional studies were identified through reference lists of included articles. The search strategy combined MeSH and free text terms. English search terms included “Pancreatitis,” “Severe Acute Pancreatitis,” “Acute Lung Injury,” “ARDS,” and “Chinese Herbal Medicine.” Chinese search terms included “acute pancreatitis,” “severe acute pancreatitis,” “acute lung injury,” “acute respiratory distress syndrome,” “traditional Chinese medicine,” and “integrative medicine.” The detailed search strategy is provided in Supplementary Table S1.

### Inclusion and exclusion criteria

Inclusion Criteria: 1) Studies involving participants aged ≥18 years 2) No restrictions based on race or gender. 3) Studies diagnosing SAP and ALI/ARDS according to established criteria. 4) RCTs published internationally that investigated the treatment of SAP-ALI/ARDS with CHM. 5) Interventions: The control group received Western medical treatment (e.g., mechanical ventilation, fasting, gastrointestinal decompression, infection control, and electrolyte balance maintenance). 6) The experimental group received CHM or compound Chinese herbal formulas in addition to conventional treatment. 7) Outcome measures included at least one of the following: clinical efficacy, mortality, pain relief time, first defecation time, bowel sounds recovery time, mechanical ventilation duration, Intensive Care Unit (ICU) stay, oxygenation index, APACHE II score, Traditional Chinese Medicine (TCM) syndrome score, TNF-α, or IL-6. 8) Baseline APACHE II score ≥ 8.

Exclusion Criteria: 1) Non-RCTs (e.g., observational or retrospective studies). 2) Studies that did not use CHM or only used non-pharmacological TCM interventions (e.g., acupuncture or massage). 3) Studies with incomplete data or unavailable full texts. 4) Studies that did not report efficacy outcomes. 5) Studies involving SAP with other severe complications (e.g., pancreatic necrosis or sepsis). 6) Duplicate publications. 7) Animal experiments or non-clinical studies.

### Data extraction and quality assessment

Three independent reviewers (XH, MZ and CD) screened the literature according to predefined eligibility criteria. Disagreements were resolved through discussion or arbitration by a third reviewer (QQN). Missing data were sought from the original authors whenever possible. Extracted information included: Basic study characteristics: First author, publication year, study population, study period, region, intervention measures, and control schemes. Outcome measures: Clinical efficacy, mortality, symptom relief time (e.g., pain relief, first defecation, bowel sounds recovery), mechanical ventilation duration, ICU stay, oxygenation index, APACHE II score, TCM syndrome score, and inflammatory biomarkers (TNF-α, IL-6).

The quality of included studies was assessed using the Cochrane Risk of Bias Tool. Two reviewers independently evaluated seven domains: random sequence generation, allocation concealment, blinding of participants and personnel, blinding of outcome assessors, completeness of outcome data, selective reporting, and other biases. Each domain was categorized as “low risk,” “unclear,” or “high risk.” Disagreements were resolved by a third reviewer.

### Statistical analysis

Meta-analysis was conducted using Rev Man 5.4 software. Heterogeneity was assessed using the I^2^ statistic. Data with low heterogeneity (I^2^ ≤ 50%, *P* > 0.10) were analyzed using a fixed-effect model, while data with high heterogeneity (I^2^ > 50%, *P* < 0.10) were analyzed using a random-effects model, followed by sensitivity analyses. Continuous variables (e.g., symptom relief time, APACHE II score, oxygenation index) were analyzed using mean differences (MD) where scales were uniform; otherwise, standardized mean differences (SMD) were applied. Binary variables (e.g., mortality, efficacy rate) were analyzed using risk ratios (RR). Effect sizes were reported with 95% confidence intervals (CI), and statistical significance was set at *P* ≤ 0.05.

## Results

### Study selection

**FIGURE 1:**
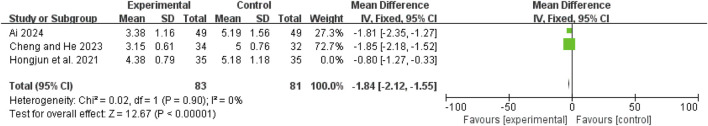
Flow chart of the literature search. CBM, Chinese Biomedical Literature Database SinoMed; CNKI, China National Knowledge Network journal full-text database; Wan Fang, Wan fang data knowledge service platform; VIP, VIP Chinese science and technology periodical database.

The initial search yielded 300 articles The distribution of studies across databases was as follows: PubMed (n = 1), Cochrane Library (n = 4), Web of Science (n = 9), Embase (n = 7), ProQuest (n = 2), OVID (n = 0), Scopus (n = 1), CBM (n = 34), CNKI (n = 42), Duxiu (n = 111), WanFang (n = 80), and VIP (n = 9). After removing duplicates, 178 articles remained. Non-RCTs (e.g., reviews, systematic reviews, and animal studies) were excluded, leaving 75 articles. After screening the abstracts, 58 studies were excluded due to mismatched interventions or outcomes, resulting in 17 articles. Following full-text assessment, one study was excluded due to unavailable full text, and three studies were excluded due to methodological inconsistencies. Ultimately, 13 RCTs were included (Ai, 2024; Chao et al., 2024; Chunyu et al., 2013; Hongjun et al., 2021; Jian-Nan et al., 2012; Li, 2014; Qiong et al., 2019; Xiao-Sheng et al., 2012; Xing et al., 2021; Xing and Kun, 2023; Yu and Fu, 2012; Cheng and He, 2023; Jie et al., 2022). The results of the literature search process are shown in Figure 1.

### Risk of bias

**FIGURE 2:**
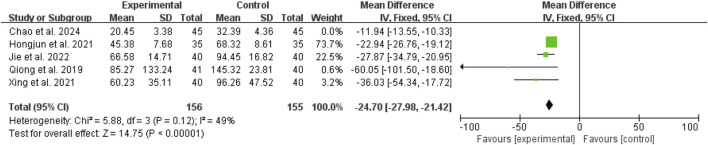
Risk of bias graph presented as percentage across all included studies.

**FIGURE 3:**
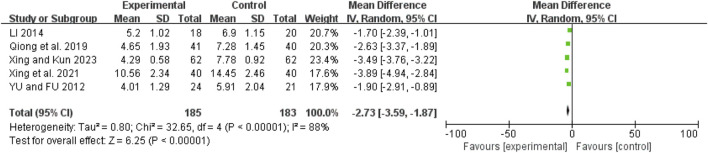
Risk of bias summary of the included studies.

The quality of the included studies was evaluated using the Cochrane Risk of Bias Tool. The results of the risk of bias assessment are presented in Figures 2, 3. The basic characteristics and intervention details of the included studies are summarized in Tables 1 and 2. The evidence of quality control of Chinese medicine prescriptions in the experimental group included in the systematic review is shown in Table 3.

**TABLE 1:** Total sample size of the included literature.

Researcher	Research time	Experimental method	Total sample size (E/C)	Age group (E/C)	Course of disease group (E/C) (d)	ApacheⅡscore (E/C)	Outcome index
Xiao-sheng et al. (2012)	2009.10 to 2012.2	RCT (random number table method)	35 (18/17)	26 to 75		≥8	2, 6, 7, 10
Yu and Fu (2012)	2010.6 to 2011.9	RCT (Drug distribution is randomized through the central randomization system of clinical studies)	45 (24/21)	54.47 ± 9.94/55.31 ± 10.94	23.97 ± 10.79/26.90 ± 12.01	11.71 ± 2.40/11.19 ± 2.33	2, 7, 8, 10
Jian-nan et al. (2012)	2009.9 to 2011.8	RCT (random assignment)	61 (30/31)	58.67 ± 16.88/54.81 ± 16.02		10.17 ± 1.21/10.52 ± 1.36	4, 7, 9
Chunyu et al. (2013)	2011.7 to 2012.8	RCT	40 (20/20)	65.7 ± 5.4/64.8 ± 5.8		11.60 ± 3.52/11.50 ± 3.61	2, 6, 7, 9
LI (2014)	2010.1 to 2012.10	RCT	38 (18/20)	44.1 ± 5.13/47.1 ± 3.71		27.18 ± 3.12/28.19 ± 5.11	2, 7, 8, 9
Qiong et al. (2019)	2016.3 to 2019.3	RCT (block randomization)	90 (45/45)	46.32 ± 9.85/45.62 ± 10.37		9.67 ± 1.25/9.23 ± 1.14	1, 3, 4, 6, 7, 8, 10, 11, 12
Xing et al. (2021)	2017.1 to 2019.11	RCT (random number generation)	80 (40/40)	37.54 ± 9.22/36.34 ± 9.59	1.89 ± 0.38/1.92 ± 0.31	11.8 ± 2.6/11.6 ± 2.4	1, 3, 4, 6, 7, 8, 10, 11
Hongjun et al. (2021)	2019.1 to 2020.10	RCT (random number table method)	70 (35/35)	49.57 ± 3.15/50.11 ± 2.98	3.15 ± 1.14/3.30 ± 1.09	33.11 ± 1.18/32.28 ± 2.11	1, 3, 5, 6, 7, 10, 11
Jie et al. (2022)	2021.1 to 2022.1	RCT (random number table method)	80 (40/40)	45.85 ± 6.78/45.26 ± 6.47	1.01 ± 0.13/0.99 ± 0.13	18.85 ± 2.14/18.64 ± 2.07	2, 3, 6, 7, 9, 10, 11, 12
Xing and Kun (2023)	2016.1 to 2021.1	RCT (block randomization)	124 (62/62)	42.70 ± 6.31/42.58 ± 6.23		21.08 ± 3.11/21.18 ± 3.15	1, 3, 4, 6, 7, 8, 9, 10, 12
Chao et al. (2024)	2022.10 to 2023.7	RCT (random number table method)	90 (45/45)	47.86 ± 5.38/47.34 ± 5.25	0.15 ± 0.04/0.16 ± 0.04	14.36 ± 2.21/14.46 ± 2.28	1, 3, 6, 11
Ai (2024)	2020.8 to 2022.11	RCT (random number table method)	98 (49/49)	47.25 ± 3.49/47.14 ± 3.26			1, 3, 5
Cheng and He (2023)	2021.10 to 2022.12	RCT (random number table method)	66 (34/32)	41.44 ± 11.51/42.94 ± 12.54		12.79 ± 2.89/11.41 ± 2.78	1, 3, 4, 5, 7, 9

E, experimental group; C, control group; d, day; 1, Clinical efficacy; 2, Mortality rate; 3, Time to abdominal pain relief; 4, Time to first bowel movement; 5, Time to bowel sound recovery; 6, Tumor necrosis factor-α; 7, Oxygenation index; 8, Mechanical ventilation duration; 9, Post-treatment APACHE II, score; 10, ICU, stay duration; 11, Interleukin-6; 12, Post-treatment TCM, syndrome score.

**TABLE 2:** Included literature interventions.

Researcher	Intervention measure		Ingredients of HM formula	TCM function (s) of HM formula (if reported)
	E	C		
Xiao-sheng et al. (2012)	The suspension of Euphorbia kansui Liou ex S.B.Ho powder was perfused into the gastric tube and WM	WM	*Euphorbia kansui Liou ex S.B.Ho powder*	Removing water retention by purgation
Yu and Fu (2012)	Qingfei Chengqi granule suspension was perfused into the gastric tube and enema and WM	Placebo was perfused into the gastric tube and enema (The Clinical Evaluation Center of China Academy of Chinese Medical Sciences distributes drugs) and WM	The composition of Chinese medicine prescriptions of Qingfei Chengqi granules: Trichosanthes kirilowii Maxim.; Pinellia ternata (Thunb.) Makino; Magnolia officinalis Rehder and E.H.Wilson; Citrus aurantium L.; Rheum palmatum L.; Coptis chinensis Franch	Clearing heat and promoting diuresis; Clearing heat-toxin
Jian-nan et al. (2012)	Laifu Chengqi decoction enema and WM	WM	The composition of Chinese medicine prescriptions of Laifu Chengqi decoction: Rheum palmatum L.; Magnolia officinalis Rehder and E.H.Wilson; Citrus aurantium L.; Mirabilite; Aucklandia lappa Decne.; Raphanus raphanistrum subsp. Sativus (L.) Domin; Achyranthes bidentata Blume	Clearing heat and purgation; Regulation qi-flowing for relieving pain
Chunyu et al. (2013)	Dahuang Fuzi decoction enema and WM	Warm soap water enema and WM	The composition of Chinese medicine prescriptions of Dahuang Fuzi decoction: Rheum palmatum L.; Aconitum carmichaelii Debeaux; Asarum heterotropoides F.Schmidt	Warming yang for relaxing bowels; Treating coagulated cold by purgation
LI (2014)	Qingyi decoction combined with Dahuang Mudan decoction nasal feeding and WM	WM	The composition of Chinese medicine prescriptions of Qingyi decoction and Dahuang Mudan decoction: Bupleurum chinense DC.; Paeonia lactiflora Pall.; Mirabilite; Citrus aurantium L.; Corydalis yanhusuo (Y.H.Chou & Chun C.Hsu) W.T.Wang ex Z.Y.Su and C.Y.Wu; Paeonia lactiflora Pall.; Paeonia × suffruticosa Andrews; *Lonicera japonica* Thunb.; Lysimachia christinae Hance; Gardenia jasminoides J.Ellis; Glycyrrhiza uralensis Fisch. exDC.; Prunus persica (L.) Batsch; Scutellaria baicalensis Georgi	Clearing heat and purgation; Clearing heat-toxin; Promoting blood circulation for removing blood stasis
Qiong et al. (2019)	Jiawei Dacheng Qi decoction retention enema and WM	WM	The composition of Chinese medicine prescriptions of Modified Dacheng Qi decoction: Rheum palmatum L.; Mirabilite; Citrus aurantium L.; Magnolia officinalis Rehder and E.H.Wilson; Prunus persica (L.) Batsch; Carthamus tinctorius L.; Cinnamomum aromaticum Nees; Bupleurum chinense DC.; Paeonia lactiflora Pall.; Aucklandia lappa Decne	Clearing heat and purgation; Regulation qi-flowing for relieving pain; Promoting blood circulation for removing blood stasis
Xing et al. (2021)	Qingyi Tongfu tiaofei yin nasal feeding and CBP and WM	CBP (Adopt high volume hemofiltration mode, blood flow rate of 200–250 mL/min,the flow rate of the replacement solution was 4–5 L/h,and local sodium citrate was used for anticoagulation) and WM	The composition of Chinese medicine prescriptions of Qingyi Tongfu tiaofei yin: Rheum palmatum L.; Gypsum; Citrus aurantium L.; Aucklandia lappa Decne.; Mirabilite; Prunus mandshurica (Maxim.) Koehne; Trichosanthes kirilowii Maxim.; Magnolia officinalis Rehder and E.H.Wilson; Corydalis yanhusuo (Y.H.Chou & Chun C.Hsu) W.T.Wang ex Z.Y.Su and C.Y.Wu; Scutellaria baicalensis Georgi; Salvia miltiorrhiza Bunge; Cyathula officinalis K.C.Kuan; Paeonia lactiflora Pall.; Coptis chinensis Franch.; Glycyrrhiza uralensis Fisch. exDC.	Clearing heat and promoting diuresis; Purging lung and purgation
Hongjun et al. (2021)	The first Qingyi Xianxiong decoction is injected through a gastric tube or jejunal nutrition tube. The second decoction of Chinese liquid enema and WM	WM	The composition of Chinese medicine prescriptions of Qingyi Xianxiong decoction: Rheum palmatum L.; Scutellaria baicalensis Georgi; Bupleurum chinense DC.; Mirabilite; Citrus aurantium L.; Paeonia lactiflora Pall.; Aucklandia lappa Decne.; Melia toosendan Siebold & Zucc.; Corydalis yanhusuo (Y.H.Chou & Chun C.Hsu) W.T.Wang ex Z.Y.Su and C.Y.Wu; Pinellia ternata (Thunb.) Makino; Euphorbia kansui Liou ex S.B.Ho	Dispersing stagnated liver qi for regulating stomach; Regulation qi-flowing for relieving pain; Clearing heat-toxin; Clearing intestinal heat for relaxing bowels
Jie et al. (2022)	Oral or nasal feeding of Maxing Shigan decoction and NIPPV and WM	NIPPV (The ventilation mode was bi-positive pressure (Bi-PAP), expiratory pressure was 4–8 cmH2O, inspiratory pressure was 8–18 cmH2O, and oxygen concentration was 30%–100%) and WM	The composition of Chinese medicine prescriptions of Maxing Shigan decoction: Ephedra sinica Stapf; Gypsum; Prunus mandshurica (Maxim.) Koehne; Citrus aurantium L.; Magnolia officinalis Rehder and E.H.Wilson; Rheum palmatum L.; Paeonia lactiflora Pall.; Glycyrrhiza uralensis Fisch. exDC.	Resolving exterior with pungent and cool natured drugs; Clearing heat and purgation
Xing and Kun (2023)	Qingyi Tongfu Tiaofei Yin and Sequential Mechanical Ventilation and WM	Sequential Mechanical Ventilation and WM	The composition of Chinese medicine prescriptions of Qingyi Tongfu Tiaofei Yin: Rheum palmatum L.; Gypsum; Aucklandia lappa Decne.; Citrus aurantium L.; Prunus mandshurica (Maxim.) Koehne; Mirabilite; Trichosanthes kirilowii Maxim.; Corydalis yanhusuo (Y.H.Chou & Chun C.Hsu) W.T.Wang ex Z.Y.Su and C.Y.Wu; Magnolia officinalis Rehder and E.H.Wilson; Salvia miltiorrhiza Bunge; Scutellaria baicalensis Georgi; Paeonia lactiflora Pall.; Cyathula officinalis K.C.Kuan; Glycyrrhiza uralensis Fisch. exDC.; Coptis chinensis Franch	Clearing heat and purgation; Regulation qi-flowing for relieving pain; Dispersing stagnated liver qi for promoting bile flow
Chao et al. (2024)	Dacheng Qi decoction nasal feeding and Octreotide injection and WM	Octreotide injection (Sinopharm One Heart Pharmaceutical Co., LTD.; H20041557; 1 mL: 0.1 mg) and WM	The composition of Chinese medicine prescriptions of Dacheng Qi decoction: Rheum palmatum L.; Mirabilite; Citrus aurantium L.; Magnolia officinalis Rehder and E.H.Wilson	Clearing heat and purgation; Drastically purging the heat accumulation
Ai (2024)	Qingyi decoction enema or gastric tube and Octreotide injection and WM	Octreotide injection (Sanning, Approval number: H20090948, Novartis Pharma Schweiz AG) and WM	The composition of Chinese medicine prescriptions of Qingyi decoction: Bupleurum chinense DC.; Paeonia lactiflora Pall.; Rheum palmatum L.; Aucklandia lappa Decne.; Mirabilite; Scutellaria baicalensis Georgi; Corydalis yanhusuo (Y.H.Chou & Chun C.Hsu) W.T.Wang ex Z.Y.Su and C.Y.Wu; Neopicrorhiza scrophulariiflora (Pennell) D.Y.Hong	Clearing heat and purgation; Tong li gong xia; Promoting blood circulation for removing blood stasis; Clearing heat and promoting diuresis
Cheng and He (2023)	Oral or gastric tube injection of Jiawei Qingyi decoction and CBP and WM	CBP (CVVH mode was adopted as the treatment mode.The replacement liquid was selected as the hemofiltration replacement base liquid--specification: 4000 mL PVC bag, manufacturer: Chengdu Qingshan Likang Pharmaceutical Co., LTD., SINopMA Approval number: H20080452) and WM	The composition of Chinese medicine prescriptions of Jiawei Qingyi decoction: Bupleurum chinense DC.; Scutellaria baicalensis Georgi; Rheum palmatum L.; Pinellia ternata (Thunb.) Makino; Citrus aurantium L.; Paeonia lactiflora Pall.; Magnolia officinalis Rehder and E.H.Wilson; Zingiber officinale Roscoe; Trichosanthes kirilowii Maxim.; Descurainia sophia (L.) Webb ex Prantl; Prunus persica (L.) Batsch; Taraxacum mongolicum Hand-Mazz.; Paeonia × suffruticosa Andrews; Angelica sinensis (Oliv.) Diels; Mirabilite; Glycyrrhiza uralensis Fisch. exDC.	Clearing heat-toxin; Promoting blood circulation for removing blood stasis; Purging lung and purgation

HM, herbal medicine; WM, western medicine; CBP, continuous blood purification; NIPPV, NonInvasive Positive Pressure Ventilation; CVVH, Continuous veno-venous hemofiltration.

**TABLE 3:** Evidence of quality control of the administered TCM in the trials included in the systematic review.

Researcher	HM formula of experimental group	Source/supplier of HM formula	Ingredients of HM formula (dosage)	Extraction method of HM formula	Treatment method	Treatment time
Xiao-sheng et al. (2012)	Euphorbia kansui Liou ex S.B.Ho powder	Guangdong Provincial Hospital of Traditional Chinese Medicine Pharmacy	*Euphorbia kansui Liou ex S.B.Ho powder (1 to 1.5g)*	The suspension was prepared with 1–1.5 g of *Euphorbia kansui Liou ex S.B.Ho* powder and 50 mL of normal saline	After 50 mL of Chinese medicinal liquid was injected into the gastric tube, the gastric tube was clamped for 30–60min	2 to 3 times/day, 5 days of treatment
Yu and Fu (2012)	Qingfei Chengqi granules	Jiangyin Tianjiang Pharmaceutical Co., LTD.	*Qingfei Chengqi granule 2 bags*	one bag of Qingfei Chengqi granules was decocted with water into 200 mL of Chinese medicine liquid and injected into the stomach tube. One bag of Qingfei Chengqi granules was decocted with water into 400 mL of Chinese medicine liquid enema	Infusion of Chinese medicine into gastric tube and enema	2 times/day, 7 days of treatment
Jian-nan et al. (2012)	Laifu Chengqi decoction	Pharmacy of Traditional Chinese Medicine, General Hospital of Tianjin Medical University	*Rheum palmatum L. (20g); Magnolia officinalis Rehder and E.H.Wilson (15g); Citrus aurantium L. (10g); Mirabilite (15g); Aucklandia lappa Decne. (10g); Raphanus raphanistrum subsp. Sativus (L.) Domin (15g); Achyranthes bidentata Blume. (10g)*	Laifu Chengqi decoction is boiled with water into 300 mL of Chinese medicinal liquid	300 mL Chinese liquid retention enema. Enema method: The patient was positioned laterally, the depth of anal tube insertion was about 20–25cm, and the juice was dropped into the intestinal cavity (20 mL/min)	2 times/day, 5 days of treatment
Chunyu et al. (2013)	Dahuang Fuzi decoction			Dahuang Fuzi decoction decocted with water into 200 mL herbal solution	200 mL Chinese liquid retention enema	2 times/day, 7 days of treatment
LI (2014)	Qingyi decoction combined with Dahuang Mudan decoction	Zhejiang Xinchang County Hospital of traditional Chinese medicine pharmacy	*Bupleurum chinense DC. (15g); Paeonia lactiflora Pall. (15g); Mirabilite (12g); Citrus aurantium L. (9g); Corydalis yanhusuo (Y.H.Chou & Chun C.Hsu) W.T.Wang ex Z.Y.Su and C.Y.Wu (9g); Paeonia lactiflora Pall. (9g); Paeonia × suffruticosa Andrews (10g); Lonicera japonica Thunb. (15g); Lysimachia christinae Hance (10g); Gardenia jasminoides J.Ellis (12g); Glycyrrhiza uralensis Fisch. exDC. (10g); Prunus persica (L.) Batsch (10g); Scutellaria baicalensis Georgi. (10g)*	Qingyi Decoction combined with Dahuang Mudan decoction are boiled in water into 300 mL Chinese medicinal liquid	Intranasal feeding with 150 mL Chinese liquid medicine	2 times/day, 7 days of treatment
Qiong et al. (2019)	Jiawei Dacheng Qi decoction	Department of Pharmacy, the First Affiliated Hospital of Hunan University of Chinese Medicine	*Rheum palmatum L. (20g); Mirabilite (10g); Citrus aurantium L. (20g); Magnolia officinalis Rehder and E.H.Wilson (20g); Prunus persica (L.) Batsch (20g); Carthamus tinctorius L. (10g); Cinnamomum aromaticum Nees (15g); Bupleurum chinense DC. (20g); Paeonia lactiflora Pall. (20g); Aucklandia lappa Decne. (20g)*	Jiawei Dachengqi decoction and boil it into 400 mL Chinese medicine liquid	200 mL Chinese liquid retention enema. Methods: The patient was lying on the left side, the depth of enema was 20–25cm, and the retention time was >30min	2 times/day, 5 days of treatment
Xing et al. (2021)	Qingyi Tongfu tiaofei yin	Zhejiang Jinhua Central Hospital pharmacy of traditional Chinese medicine	*Rheum palmatum L. (12g); Gypsum (12g); Citrus aurantium L. (12g); Aucklandia lappa Decne. (12g); Mirabilite (10g); Prunus mandshurica (Maxim.) Koehne (10g); Trichosanthes kirilowii Maxim. (10g); Magnolia officinalis Rehder and E.H.Wilson (15g); Corydalis yanhusuo (Y.H.Chou & Chun C.Hsu) W.T.Wang ex Z.Y.Su and C.Y.Wu (15g); Scutellaria baicalensis Georgi (15g); Salvia miltiorrhiza Bunge (15g); Cyathula officinalis K.C.Kuan (15g); Paeonia lactiflora Pall. (15g); Coptis chinensis Franch. (8g); Glycyrrhiza uralensis Fisch. exDC. (6g)*	Qingyitongfu tiaofei decoction with water decocted into 200 mL Chinese liquid medicine	Intranasal feeding with 100 mL Chinese liquid	2 times/day, 10 days of treatment
Hongjun et al. (2021)	Qingyi Xianxiong decoction	Shandong Rizhao City Hospital of traditional Chinese medicine pharmacy	*Rheum palmatum L.; Scutellaria baicalensis Georgi; Bupleurum chinense DC.; Mirabilite; Citrus aurantium L.; Paeonia lactiflora Pall.; Aucklandia lappa Decne.; Melia toosendan Siebold & Zucc.; Corydalis yanhusuo (Y.H.Chou & Chun C.Hsu) W.T.Wang ex Z.Y.Su and C.Y.Wu; Pinellia ternata (Thunb.) Makino; Euphorbia kansui Liou ex S.B.Ho. (The specific dosage is not specified in the literature)*	Qingyi Xianxiong decoction with water decocted into 400 mL of herbal liquid	The first boiled 200 mL of Chinese medicine liquid was injected through the gastric tube/jejunal nutrition tube, and the gastric tube was closed for 1 h after injection. The second decocted 200 mL Chinese medicinal liquid enema	Gastric tube/jejunal nutrition tube injection: 2 times/day; Enema:1 time/day; 14 days of treatment
Jie et al. (2022)	Maxing Shigan decoction	Hubei Maternal and Child Health Hospital pharmacy of traditional Chinese medicine	*Ephedra sinica Stapf (10g); Gypsum (30g); Prunus mandshurica (Maxim.) Koehne (12g); Citrus aurantium L. (15g); Magnolia officinalis Rehder and E.H.Wilson (15g); Rheum palmatum L. (15g); Paeonia lactiflora Pall. (12g); Glycyrrhiza uralensis Fisch. exDC. (6g)*	Decocting Ma Xing Shigan decoction with water into 100 mL of Chinese medicinal liquid	Warm feeding/nasal feeding	2 times/day, 7 days of treatment
Xing and Kun (2023)	Qingyi Tongfu Tiaofei Yin	Pharmacy of Traditional Chinese Medicine, Jinhua Hospital, Zhejiang University School of Medicine	*Rheum palmatum L. (12g); Gypsum (12g); Aucklandia lappa Decne. (12g); Citrus aurantium L. (12g); Prunus mandshurica (Maxim.) Koehne (10g); Mirabilite (10g); Trichosanthes kirilowii Maxim. (10g); Corydalis yanhusuo (Y.H.Chou & Chun C.Hsu) W.T.Wang ex Z.Y.Su and C.Y.Wu (15g); Magnolia officinalis Rehder and E.H.Wilson (15g); Salvia miltiorrhiza Bunge (15g); Scutellaria baicalensis Georgi (15g); Paeonia lactiflora Pall. (15g); Cyathula officinalis K.C.Kuan (15g); Glycyrrhiza uralensis Fisch. exDC. (6g); Coptis chinensis Franch. (8g)*	Qingyitongfu tiaofei decoction with water decocted into 200 mL Chinese liquid medicine		2 times/day, 14 days of treatment
Chao et al. (2024)	Dacheng Qi decoction	Shandong Rizhao City People’s Hospital and Rizhao City Hospital of Traditional Chinese medicine pharmacy	*Rheum palmatum L. (10g); Mirabilite (20g); Citrus aurantium L. (15g); Magnolia officinalis Rehder and E.H.Wilson. (15g)*	Dachengqi decoction is decocted with water into 200 mL Chinese medicinal liquid	Intranasal feeding with 100 mL Chinese liquid	2 times/day, 14 days of treatment
Ai (2024)	Qingyi decoction	Anqing Hospital of Traditional Chinese Medicine Pharmacy, Anhui province	*Bupleurum chinense DC. (15g); Paeonia lactiflora Pall. (15g); Rheum palmatum L. (15g); Aucklandia lappa Decne. (9g); Mirabilite (9g); Scutellaria baicalensis Georgi (9g); Corydalis yanhusuo (Y.H.Chou & Chun C.Hsu) W.T.Wang ex Z.Y.Su and C.Y.Wu (9g); Neopicrorhiza scrophulariiflora (Pennell) D.Y.Hong. (9g)*	Qingyi Decoction is decocted with water into 250 mL Chinese liquid	During the fasting period, the patient received 200 mL enema and 50 mL gastric tube infusion after open diet.	2 times/day, 14 days of treatment
Cheng and He (2023)	Jiawei Qingyi decoction	The First Clinical Medical College of Yunnan University of Traditional Chinese Medicine Pharmacy	*Bupleurum chinense DC. (20g); Scutellaria baicalensis Georgi (15g); Rheum palmatum L. (12g); Pinellia ternata (Thunb.) Makino (15g); Citrus aurantium L. (15g); Paeonia lactiflora Pall. (20g); Magnolia officinalis Rehder and E.H.Wilson (15g); Zingiber officinale Roscoe (10g); Trichosanthes kirilowii Maxim. (30g); Descurainia sophia (L.) Webb ex Prantl (15g); Prunus persica (L.) Batsch (15g); Taraxacum mongolicum Hand-Mazz. (20g); Paeonia × suffruticosa Andrews (15g); Angelica sinensis (Oliv.) Diels (15g); Mirabilite (9g); Glycyrrhiza uralensis Fisch. exDC. (6g)*	Jiawei Qingyi decoction and boil it into 600 mL Chinese medicine liquid	150 mL of Chinese medicine liquid is taken orally/injected into the gastric tube, and the gastric tube is clamped for 1 h after taking the medicine	2 times/day, 14 days of treatment

HM, herbal medicine.

### Random sequence generation

Six studies (Ai, 2024; Chao et al., 2024; Cheng and He, 2023; Hongjun et al., 2021; Jie et al., 2022; Xiao-Sheng et al., 2012) utilized random number tables for randomization, two studies (Qiong et al., 2019; Xing and Kun, 2023) employed block randomization techniques, one study (Jian-Nan et al., 2012) implemented random assignment, another study (Xing et al., 2021) employed random number generation, and one study (Yu and Fu, 2012) used the centralized randomization and drug distribution system from the Chinese Academy of Medical Sciences Clinical Trial Center. Additionally, two studies (Chunyu et al., 2013; Li, 2014) did not clearly describe their randomization methods, which may impact the validity of their reported outcomes.

### Allocation concealment

Except for one study (Yu and Fu, 2012), which mentioned the use of a centralized randomization system for drug allocation, the remaining 12 studies did not provide detailed information on allocation concealment and were therefore assessed as having an unclear risk.

### Blinding of participants and personnel

Regarding the blinding method, only one study (Yu and Fu, 2012) explicitly reported the use of a double-blind design, leading to a low-risk assessment. The remaining 12 studies did not mention the blinding of subjects, investigators, or outcome assessors. Considering the challenges in blinding both subjects and investigators, the absence of blinding may significantly affect the results, such as the time for symptoms (e.g., abdominal pain, nausea, vomiting, dyspnea) to resolve and the length of hospital stay. Consequently, the lack of blinding for subjects and intervention providers was judged to present a high risk.

### Blinding of outcome assessment

Only one article (Yu and Fu, 2012) explicitly stated that the research method employed was a double-blind clinical design, resulting in a low-risk assessment. However, the remaining 12 studies did not mention whether the measurement of outcomes was influenced by the lack of blinding, leading to uncertainty regarding the potential risk associated with not blinding the outcome assessors.

### Incomplete outcome data

Four studies (Li, 2014; Qiong et al., 2019; Yu and Fu, 2012; Cheng and He, 2023) reported sample dropouts or deaths, with explicit explanations provided for the dropouts. The remaining nine studies were judged to have low risk.

### Selective reporting

One study (Xing and Kun, 2023) was assessed as having a high risk because it did not adhere to predefined analysis plans. One study (Li, 2014) was deemed to have an unclear risk due to the absence of predefined outcome measures. The remaining studies were judged to have low risk.

### Other bias

All studies were assessed as having an unclear risk due to insufficient information.

## Meta-analysis results

### Clinical efficacy

**FIGURE 4:**
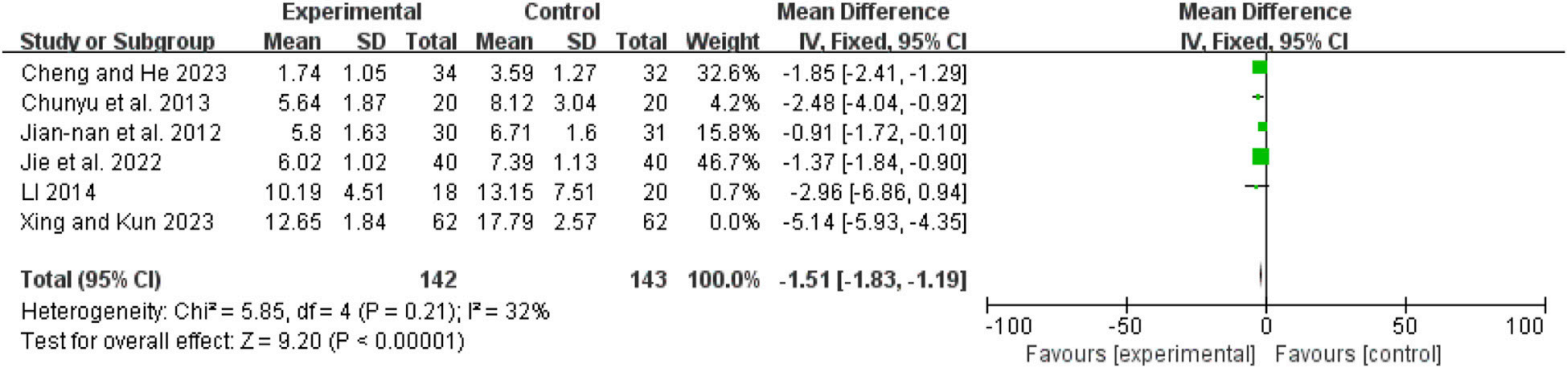
Forest map comparison of clinical efficacy between two groups.

Seven studies (Ai, 2024; Chao et al., 2024; Hongjun et al., 2021; Qiong et al., 2019; Xing et al., 2021; Xing and Kun, 2023; Cheng and He, 2023) demonstrated low heterogeneity (I^2^ = 0%, *P* = 0.65). The pooled RR was 1.26 (95% CI: 1.17 to 1.37; Z = 5.90, *P* < 0.00001), indicating that the combination of CHM and conventional WM was more effective than WM alone (Figure 4).

### Time to abdominal pain relief

**FIGURE 5:**
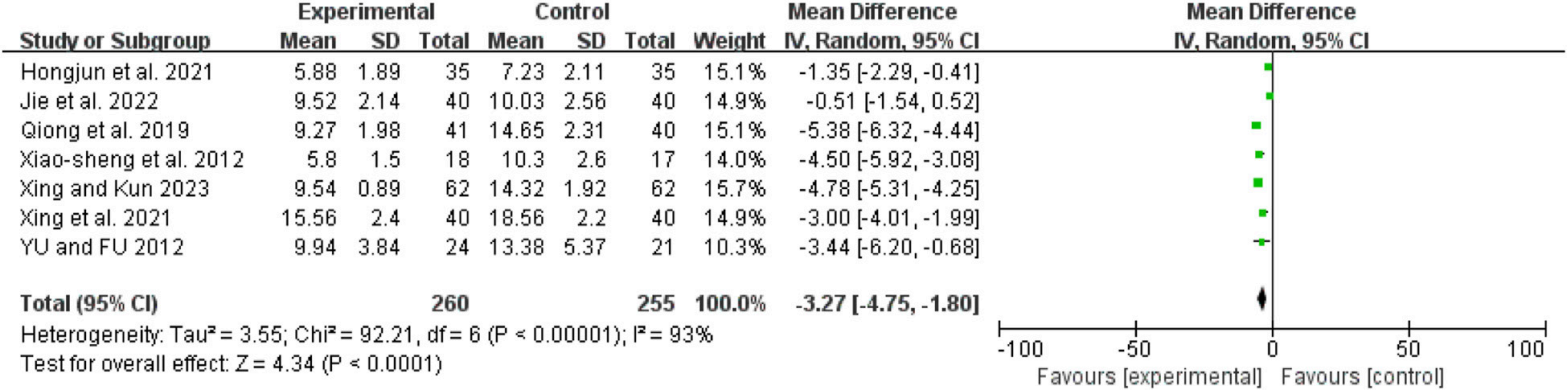
Forest map comparison of time to abdominal pain relief between two groups.

Eight studies (Ai, 2024; Chao et al., 2024; Hongjun et al., 2021; Jie et al., 2022; Qiong et al., 2019; Xing et al., 2021; Xing and Kun, 2023; Cheng and He, 2023) exhibited high heterogeneity (I^2^ = 97%, *P* < 0.00001). A random-effects model showed a mean difference (MD) of −1.56 days (95% CI: −2.14 to −0.99; Z = 5.35, *P* < 0.00001), indicating that pain relief was significantly faster in the combination therapy group compared to the WM only group (Figure 5).

### Time to first defecation

**FIGURE 6:**
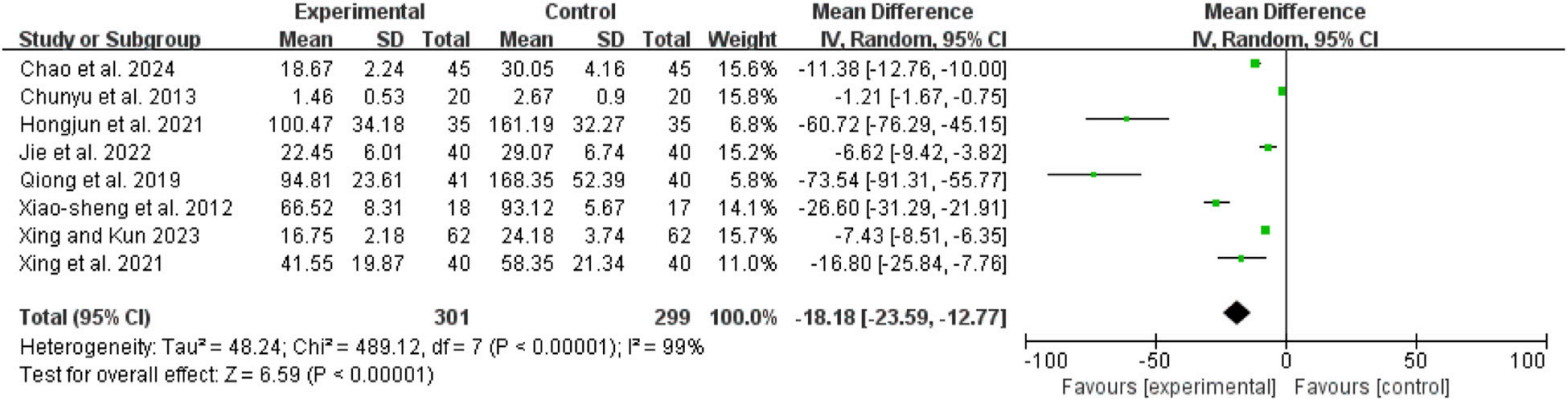
Forest map comparison of time to first bowel movement between two groups.

The initial analysis of five studies (Jian-Nan et al., 2012; Qiong et al., 2019; Xing et al., 2021; Xing and Kun, 2023; Cheng and He, 2023) revealed high heterogeneity (I^2^ = 80%, *P* = 0.0005). After excluding Shao 2023 (Xing and Kun, 2023), heterogeneity decreased (I^2^ = 39%, *P* = 0.18). The remaining four studies (Jian-Nan et al., 2012; Qiong et al., 2019; Xing et al., 2021; Cheng and He, 2023) were analyzed using a fixed-effect model, yielding an MD of −1.49 days (95% CI: −1.61 to −1.37; Z = 24.07, *P* < 0.00001), indicating that the time to first defecation was significantly shorter in the combination therapy group (Figure 6).

### Time to bowel sound recovery

**FIGURE 7:**
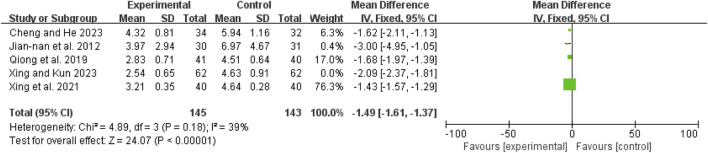
Forest map comparison of time to bowel sound recovery between two groups.

Three studies (Ai, 2024; Hongjun et al., 2021; Cheng and He, 2023) showed high heterogeneity (I^2^ = 85%, *P* = 0.001). After excluding Lu 2021 (Hongjun et al., 2021), the remaining two studies (Ai, 2024; Cheng and He, 2023) had no heterogeneity (I^2^ = 0%, *P* = 0.90). A fixed-effect model revealed an MD of −1.84 days (95% CI: −2.12 to −1.55; Z = 12.67, *P* < 0.00001), indicating faster recovery of bowel sounds in the combination therapy group (Figure 7).

### Duration of mechanical ventilation

**FIGURE 8:**
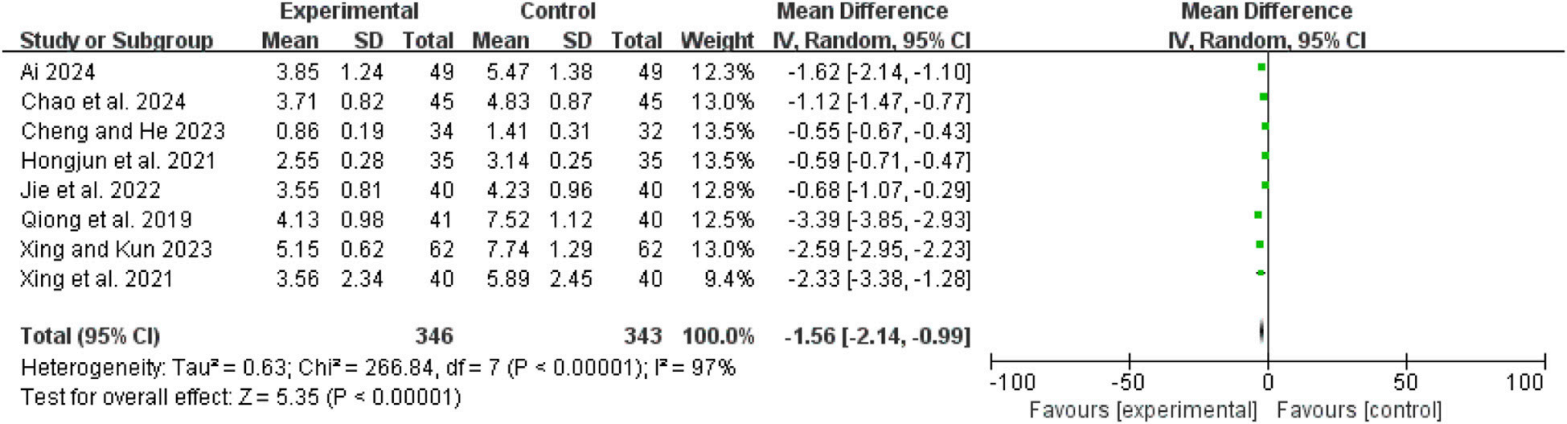
Forest map comparison of mechanical ventilation duration between two groups.

Five studies (Li, 2014; Qiong et al., 2019; Xing et al., 2021; Xing and Kun, 2023; Yu and Fu, 2012) exhibited high heterogeneity (I^2^ = 88%, *P* < 0.00001). A random-effects model showed an MD of −2.73 days (95% CI: −3.59 to −1.87; Z = 6.25, *P* < 0.00001), indicating that mechanical ventilation duration was significantly shorter in the combination therapy group (Figure 8).

### Oxygenation index

**FIGURE 9:**
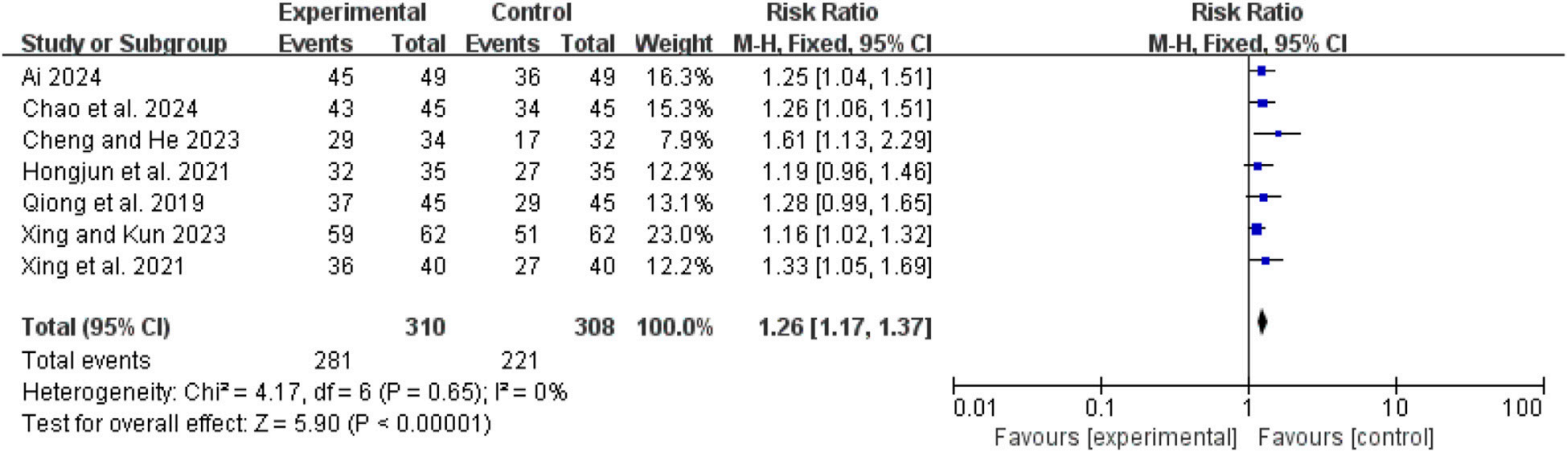
Forest map comparison of oxygenation index between two groups.

Eleven studies (Chunyu et al., 2013; Hongjun et al., 2021; Jian-Nan et al., 2012; Jie et al., 2022; Li, 2014; Qiong et al., 2019; Xiao-Sheng et al., 2012; Xing et al., 2021; Xing and Kun, 2023; Yu and Fu, 2012; Cheng and He, 2023) showed high heterogeneity (I^2^ = 93%, *P* < 0.00001). A random-effects model revealed a mean difference (MD) of 43.68 (95% CI: 27.21 to 60.14; Z = 5.20, *P* < 0.00001), indicating that the oxygenation index was significantly improved in the combination therapy group (Figure 9).

### Mortality rate

**FIGURE 10:**
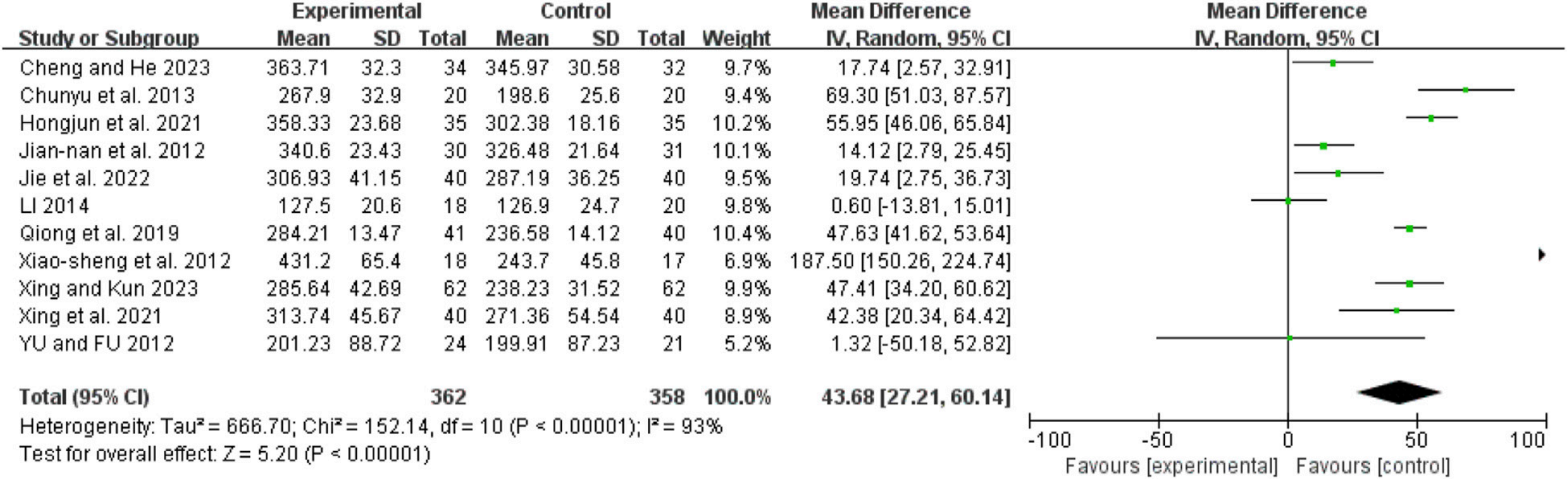
Forest map comparison of mortality rate between two groups.

Five studies (Jie et al., 2022; Li, 2014; Xiao-Sheng et al., 2012; Yu and Fu, 2012; Qiong et al., 2019) showed no heterogeneity (I^2^ = 0%, *P* = 0.96). The pooled RR was 0.47 (95% CI: 0.20 to 1.10; Z = 1.74, *P* = 0.08), with no significant difference between groups in subgroup analyses (Figure 10).

### TNF-α

**FIGURE 11:**
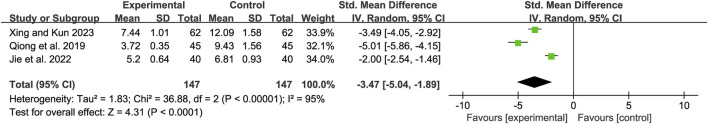
Forest map comparison of TNF-α between two groups. TNF-α, Tumor Necrosis Factor-α.

Eight studies (Chunyu et al., 2013; Hongjun et al., 2021; Jie et al., 2022; Qiong et al., 2019; Xiao-Sheng et al., 2012; Xing et al., 2021; Chao et al., 2024; Xing and Kun, 2023) exhibited high heterogeneity (I^2^ = 99%, *P* < 0.00001). A random-effects model showed a mean difference (MD) of −18.18 pg/mL (95% CI: −23.59 to −12.77; Z = 6.59, *P* < 0.00001), indicating that TNF-α levels were significantly lower in the combination therapy group (Figure 11).

### IL-6

**FIGURE 12:**
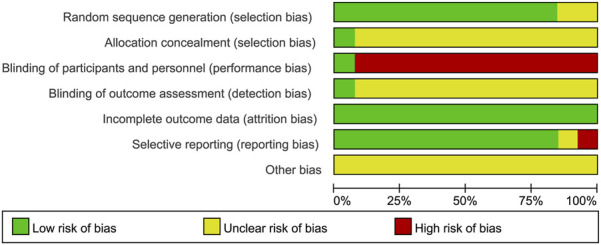
Forest map comparison of IL-6 between two groups. IL-6, Interleukin-6.

Five studies (Qiong et al., 2019; Xing et al., 2021; Chao et al., 2024; Hongjun et al., 2021; Jie et al., 2022) showed high heterogeneity (I^2^ = 92%, *P* < 0.00001). After excluding Xu 2024 (Chao et al., 2024), the remaining four studies (Hongjun et al., 2021; Jie et al., 2022; Qiong et al., 2019; Xing et al., 2021) had reduced heterogeneity (I^2^ = 49%, *P* = 0.12). A fixed-effect model revealed a mean difference (MD) of −24.70 pg/mL (95% CI: −27.98 to −21.42; Z = 14.75, *P* < 0.00001), indicating that IL-6 levels were significantly lower in the combination therapy group (Figure 12).

### Post-treatment Apache II score

**FIGURE 13:**
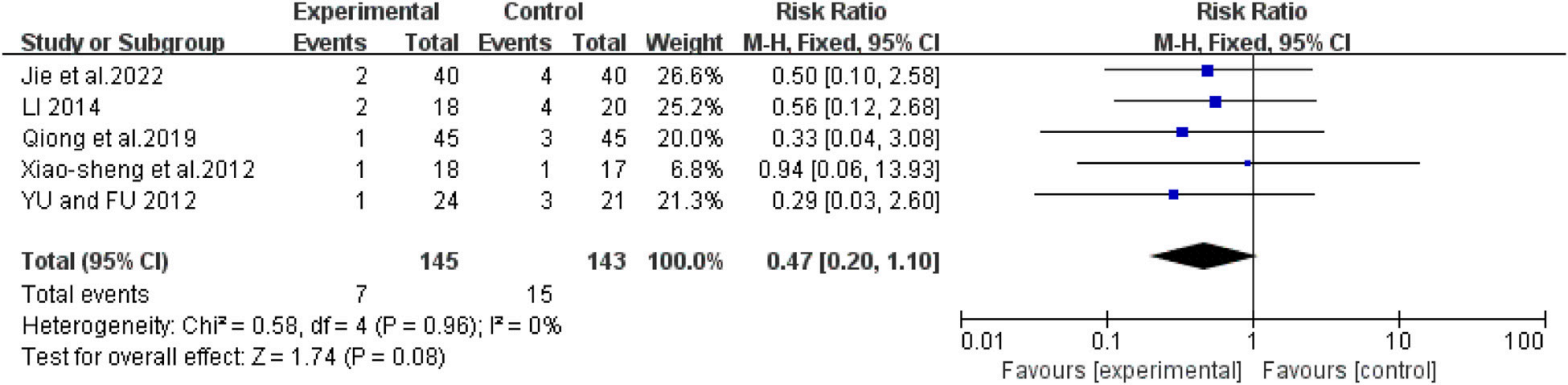
Forest map comparison of post-treatment APACHE II score between two groups.

Six studies (Chunyu et al., 2013; Jian-Nan et al., 2012; Jie et al., 2022; Li, 2014; Xing and Kun, 2023; Cheng and He, 2023) exhibited high heterogeneity (I^2^ = 93%, *P* < 0.00001). After excluding Shao 2023 (Xing and Kun, 2023), the remaining five studies (Chunyu et al., 2013; Jian-Nan et al., 2012; Jie et al., 2022; Li, 2014; Cheng and He, 2023) had no heterogeneity (I^2^ = 32%, *P* = 0.21). A fixed-effect model showed a mean difference (MD) of −1.51 (95% CI: −1.83 to −1.19; Z = 9.20, *P* < 0.00001), indicating that APACHE II scores were significantly lower in the combination therapy group (Figure 13).

### ICU stay duration

**FIGURE 14:**
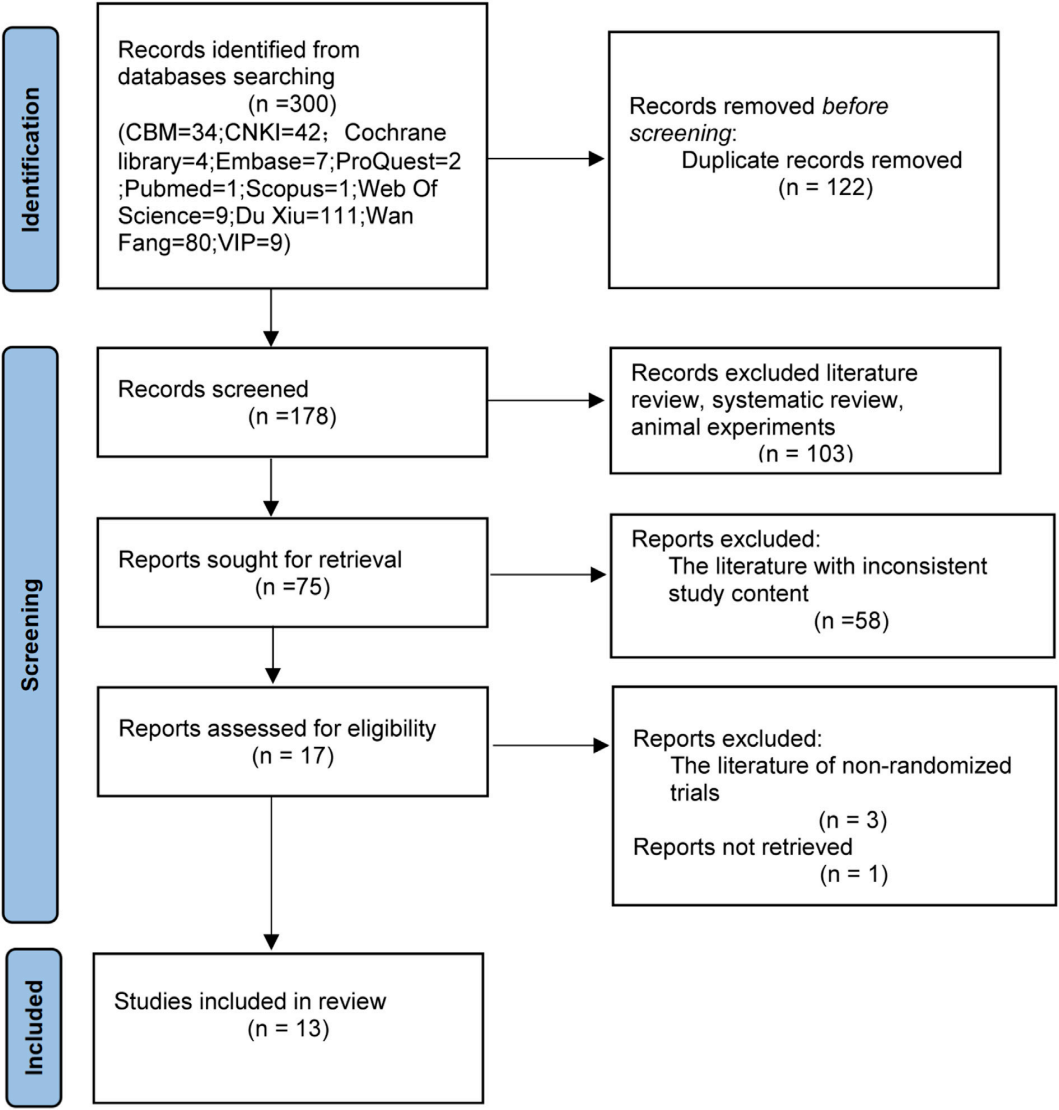
Forest map comparison of ICU stay duration between two groups. ICU, Intensive Care Unit.

Seven studies (Hongjun et al., 2021; Jie et al., 2022; Qiong et al., 2019; Xiao-Sheng et al., 2012; Xing et al., 2021; Xing and Kun, 2023; Yu and Fu, 2012) exhibited high heterogeneity (I^2^ = 93%, *P* < 0.00001). A random-effects model showed a mean difference (MD) of −3.27 days (95% CI: −4.75 to −1.80; Z = 4.34, *P* < 0.0001), indicating that ICU stay duration was significantly shorter in the combination therapy group (Figure 14).

### Post-treatment TCM syndrome score

**FIGURE 15:**
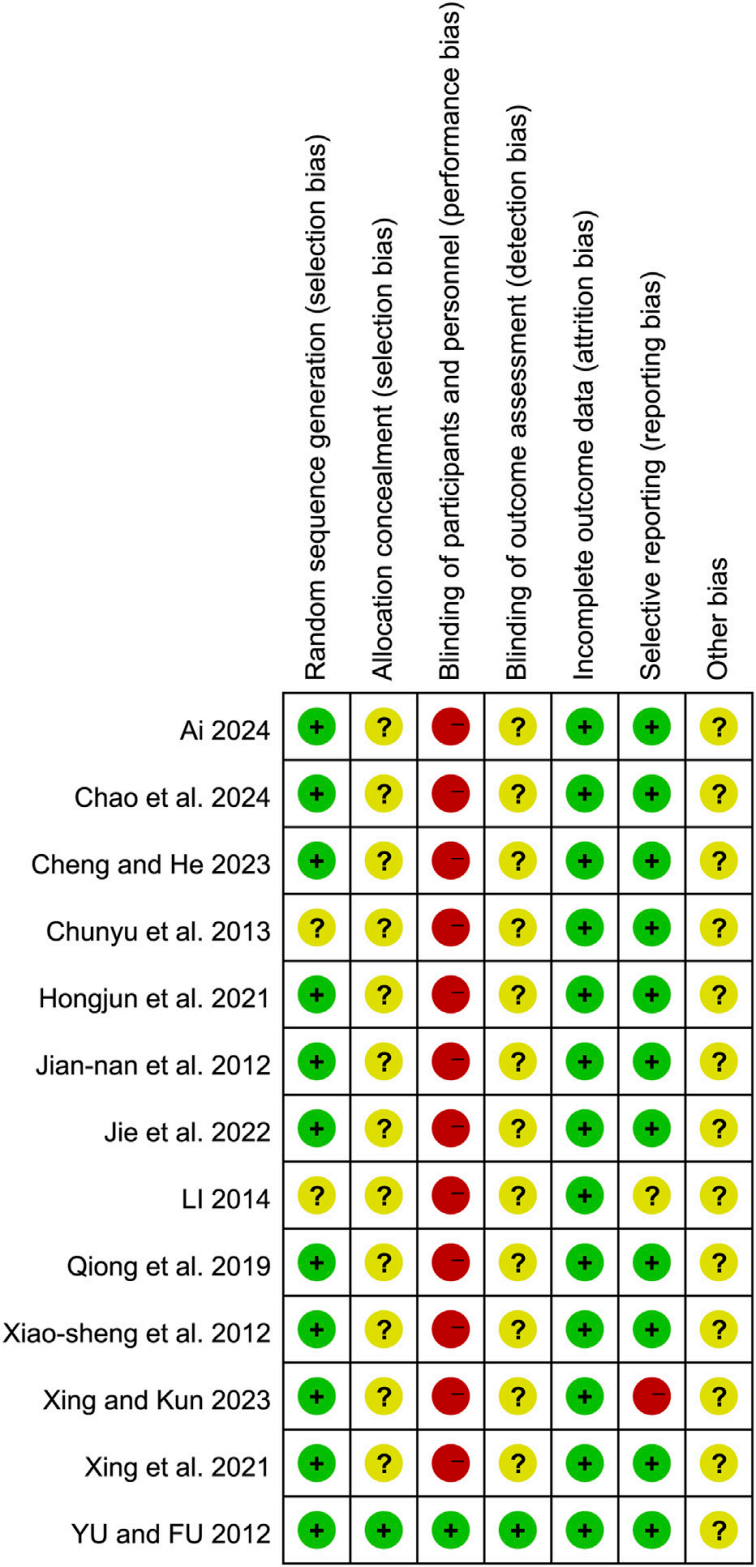
Forest map comparison of TCM Syndrome valuation Score between two groups. TCM, Traditional Chinese Medicine.

Three studies (Xing and Kun, 2023; Qiong et al., 2019; Jie et al., 2022) showed high heterogeneity (I^2^ = 95%, *P* < 0.00001). A random-effects model revealed a mean difference (MD) of −3.47 (95% CI: −5.04 to −1.89; Z = 4.31, *P* < 0.0001), indicating that TCM syndrome scores were significantly lower in the combination therapy group (Figure 15).

### Sensitivity analysis

The results for time to pain relief, TNF-α levels, oxygenation index, mechanical ventilation duration, and ICU stay duration remained stable after sequentially excluding individual studies. For time to first defecation, bowel sounds recovery, IL-6 levels, and APACHE II scores, heterogeneity was resolved after excluding outlier studies (Xing and Kun, 2023; Hongjun et al., 2021; Chao et al., 2024), allowing the use of a fixed-effect model.

### Publication bias

A funnel plot for the oxygenation index (Chunyu et al., 2013; Hongjun et al., 2021; Jian-Nan et al., 2012; Jie et al., 2022; Li, 2014; Qiong et al., 2019; Xiao-Sheng et al., 2012; Xing et al., 2021; Xing and Kun, 2023; Yu and Fu, 2012; Cheng and He, 2023) showed symmetric distribution of data points, suggesting a low risk of publication bias (Supplementary Figure S1).

## Discussion

Existing meta-analyses have primarily focused on evaluating the efficacy of TCM formulas in treating acute pancreatitis (AP) or SAP (Guo et al., 2023), or exploring the role of TCM in intervening in ALI or ARDS (Guo et al., 2021). However, there is limited systematic research on the clinical benefits of integrated Chinese and WM in treating SAP-related ALI/ARDS. This study is the first to evaluate the efficacy of such integrated therapies through a meta-analysis, incorporating evidence from 13 RCTs. The results demonstrate that the combination of Chinese herbal compound therapy with conventional WM significantly alleviates SAP-ALI/ARDS related injury and improves clinical outcomes. Compared to WM alone, the integrated treatment group showed significant reductions in pain relief time, first defecation time, bowel sounds recovery time, mechanical ventilation duration, and ICU stay duration. Additionally, the combination therapy improved the oxygenation index and reduced levels of inflammatory biomarkers (TNF-α, IL-6), as well as APACHE II scores and TCM syndrome scores.

Five core TCM treatment strategies were identified in the included studies: Clear heat and promote bowel movements, Warm yang and dissipate cold, Promote bowel movements and expel water, Release the exterior and purge the interior, and Clear heat in the liver and gallbladder. The most frequently used strategy was Clear heat and promote bowel movements, represented by formulas such as Qingfei Chengqi Granules, Laifu Chengqi Decoction, and Da Chengqi Decoction. Warm yang and dissipate cold was typified by Dahuang Fuzi Decoction, while Promote bowel movements and expel water utilized drug like Euphorbia kansui. Release the exterior and purge the interior was represented by Maxing Shigan Decoction, and six studies (Li, 2014; Hongjun et al., 2021; Xing et al., 2021; Ai, 2024; Cheng and He, 2023; Xing and Kun, 2023) employed a strategy that combines the methods of clearing liver and gallbladder heat with purging heat and promoting bowel movements, using Qingyi Decoction as the representative formula (see Tables 2 and 3 for details). Note: The treatment principles/methods and therapies related to TCM in this chapter are derived from the WHO international standard terminologies on TCM.

Clear heat and promote bowel movements, Warm yang and dissipate cold, and Promote bowel movements and expel water are categorized under the broader TCM concept of Purgative methods, which focuses on the use of purgative medications to eliminate intestinal stagnation. These subcategories are distinguished based on their mechanisms of action and clinical applications.

Clear heat and promote bowel movements is a treatment method to clear heat, reduce fire and unblock the bowel. It is indicated for heat accumulating in the large intestine. Commonly used medications include Rheum palmatum and Citrus aurantium. Classical formulas such as Da Chengqi Decoction have been shown to reduce intestinal inflammatory factors, lower serum amylase levels, and alleviate abdominal pressure while protecting the intestinal barrier (Huo et al., 2022; Liu et al., 2023; Zhu et al., 2021; Huang et al., 2012). In rat models, Zhao et al. demonstrated that Da Chengqi Decoction significantly reduced the expression of pro-inflammatory cytokines such as IL-1 mRNA and IL-6 mRNA in lung tissue (Zhao et al., 2011). Four studies (Chao et al., 2024; Jian-Nan et al., 2012; Qiong et al., 2019; Yu and Fu, 2012) combined purgative formulas with lung-protective herbs like Trichosanthes kirilowii and Pinellia ternata to treat SAP-related lung injury. Dahuang Mudan Decoction has been shown to inhibit the release of TNF-α, IL-6, and IL-8, reduce the migration of intestinal toxins, and suppress pancreatic enzyme activity (Siyuan et al., 2024; Yanzhe, 2021). Animal studies further indicate that this decoction improves lung inflammation and cell apoptosis by regulating PI3K-AKT gene expression (Dan et al., 2022).

Warm yang and dissipate cold is a treatment method to combine purgative, yang warming and cold-dissipating medicines. It is indicated for conditions due to excessive cold retention. This approach combines warming drugs (Aconitum carmichaeli and Asarum) with purgative medications like Rheum palmatum. One study (Chunyu et al., 2013) found that Dahuang Fuzi Decoction inhibits NF-κB activation, downregulates pro-inflammatory cytokines such as TNF-α and IL-1β, and alleviates SAP-related lung injury by halting disease progression.

Promote bowel movements and expel water is a treatment method to use purgative medicines to resolve water retention. It is indicated for conditions due to internal retention of water fluid. This therapy is effective in treating various acute abdominal syndromes caused by the accumulation of fluids and qi stagnation. Herbs like Euphorbia kansui is utilized in this therapy to promote diuresis and eliminate phlegm, helping to expel excess fluids through the large intestine. This approach aligns with the TCM principle that associates the lungs with the large intestine, reflecting the theory of “the lungs connect with the large intestine, and the intestine is the organ of transmission.” It embodies the TCM concept of the interrelationship between the lungs and the large intestine. Modern research indicates that these herbs may alleviate pulmonary fibrosis and reduce lung tissue inflammation (Xiaoting, 2022).

Release the exterior and purge the interior is a treatment method to combine purgative and exterior-releasing medicines. It is indicated for excess pattern involving both the exterior and interior. The classical formula Maxing Shigan Decoction exemplifies this approach, adhering to the “junior, minister, assistant, and servant” principle: Ephedra sinica (junior) induces sweating to resolve the exterior, Gypsum (minister) clears lung heat, Prunus mandshurica (assistant) lowers lung qi, and Glycyrrhiza uralensis (servant) harmonizes the effects of the other herbs. This combination achieves both exterior and interior resolution, improving lung function and alleviating dyspnea. Modern studies suggest that Maxing Shigan Decoction may modulate intestinal microbiota, influencing metabolic pathways such as palmitic acid, malic acid, and phosphate production, thereby mediating its therapeutic effects on lung injury (Zhao Jiayao, 2024).

The combination treatment of Clear heat in the liver and gallbladder and Clear heat and promote bowel movements is a specialized TCM intervention targeting the dual pathophysiological mechanisms of “shaoyang dysfunction” and “yangming intestinal heat obstruction” in the context of acute pancreatitis. The representative formula for this approach is Qingyi Decoction. The core herbal pair for this strategy is Bupleurum chinense and Scutellaria baicalensis, which work synergistically to resolve gallbladder stagnation and clear intestinal heat. Purgative combinations, such as Rheum palmatum and Mirabilite, enhance intestinal motility and bile secretion, reducing toxin accumulation and suppressing systemic inflammation. As the representative formula for the combined therapy of Clear heat in the liver and gallbladder and Clear heat and promote bowel movements, Qingyi Decoction has been shown to activate the Wnt/β-catenin signaling pathway, inhibit apoptosis of alveolar epithelial cells, and play a critical role in lung repair following lung injuries related to SAP (Yin et al., 2024).

The high application rate of Purgative methods interventions in this study substantiates the core therapeutic principle of TCM, “promoting Qi in the viscera leads to the regulation of Qi in the lungs.” This gut-targeted therapeutic strategy for respiratory conditions aligns with modern medical “gut-lung axis” theory, as SAP can disrupt gut microbiota, leading to dual mechanical and immune barrier damage in the intestinal mucosa. This disruption facilitates the mislocation of endotoxins and microbial metabolites via the portal venous system, activating inflammatory signaling pathways such as TLR4/NF-κB. These pathways trigger systemic inflammatory cascades, exacerbating alveolar-capillary barrier dysfunction (Kahalehili et al., 2020; Yang et al., 2019; Dickson et al., 2016; Wang et al., 2022a; Espirito et al., 2021; Junxi et al., 2023; Wang et al., 2022b). The “pancreas-intestine-inflammation-endotoxin-lung” pathological cascade model proposed by the Ge team further elucidates this process, involving abnormal activation of pancreatic enzymes, dysregulated intestinal microbial metabolism, and systemic systemic inflammatory response syndrome (Ge et al., 2020). One study demonstrates the potential of TCM in modulating multiple key signaling pathways, such as NF-κB, MAPK, JAK2/STAT3, NLRP3 inflammasome, Notch, Rho/ROCK, and Nrf2/ARE, in reducing inflammation, suppressing oxidative stress, and inhibiting apoptosis (Huan Chen, 2023).

Notably, this meta-analysis included 36 traditional Chinese herbal medications, with a significant proportion being laxative-type drugs (Supplementary Figure S2). Among these, Rheum palmatum was frequently used. Emodin, a primary active component of Rheum palmatum, exhibits multi-mechanistic effects in treating SAP-associated ALI. It regulates long non-coding RNA (lncRNA)-mRNA networks, modulating the expression of inflammatory genes (e.g., TNF-α, IL-6) and tissue repair genes (e.g., HO-1) to achieve its anti-inflammatory and protective effects (Xu et al., 2021). Experimental studies have demonstrated that emodin alleviates ALI associated with SAP by downregulating PBEF expression, promoting polymorphonuclear neutrophil apoptosis, and inhibiting NLRP3 inflammasome-mediated neutrophil recruitment (Cui et al., 2017; Jiang et al., 2021). Clinical evidence indicates that formulations combining Rheum palmatum, Mirabilite and Citrus aurantium demonstrate remarkable efficacy in improving oxygenation indices and reducing inflammatory markers in SAP-related ALI/ARDS (Zhao et al., 2015).

From a mechanistic perspective, the traditional Chinese medical approach of “treating the lungs by regulating the intestines” aligns with modern “gut-lung axis” theory (Yue et al., 2023). This study confirms that purgative medications modulate gut microbiota, reducing the overgrowth of harmful bacteria and the release of inflammatory substances such as lipopolysaccharides and peptidoglycans. These substances, when circulating through the gut-lung axis, stimulate inflammatory and immune responses in the lungs. Additionally, purgative drugs promote the growth of beneficial bacteria, enhance intestinal barrier function, and block pathogen translocation, effectively interrupting pathological communication along the “gut-lung axis” and mitigating pulmonary diseases (Zhu Jie, 2024). This “treat the lungs by treating the intestines” paradigm provides a molecular biological interpretation of traditional Chinese viscera-related theories. By systematically integrating traditional and modern medical frameworks, this study reveals the deep interplay between traditional medical practices and modern mechanistic research in the context of SAP-associated multi-organ dysfunction, laying a theoretical foundation for the development of innovative integrative therapeutic strategies.

This systematic review and meta-analysis provides a novel contribution to the existing literature by evaluating the efficacy of CHM combined with WM for treating SAP-related ALI/ARDS. Compared to previous meta-analyses, such as those focusing on Chinese medicine injections for ALI/ARDS or Chengqi-series decoctions for SAP, this study offers a more comprehensive approach by integrating CHM with conventional Western medical treatments. While earlier studies demonstrated the potential benefits of individual Chinese medicine injections or Chengqi-series decoctions, they were limited to specific interventions and did not explore the synergistic effects of combined therapies. For instance, Jie Guo et al. (Guo et al., 2021) literature focused on network meta-analysis of Chinese medicine injections, highlighting Xuebijing and Tanreqing as effective adjuvant treatments, but it did not address the integration of CHM with WM. Similarly, Juan Lin et al. (Lin et al., 2023) literature on Chengqi-series decoctions for SAP showed improved clinical outcomes, but it did not extend to SAP-related ALI/ARDS or the combination of CHM with Western interventions.

Our study builds on these findings by addressing the clinical efficacy of CHM-WM combination therapy, demonstrating significant improvements in disease progression indicators, oxygenation index, and inflammatory biomarkers. The results align with previous studies in showing reduced mechanical ventilation duration and improved APACHE II scores but extend these findings to the specific context of SAP-ALI/ARDS. A key innovation of this study is its focus on the gut-lung axis, providing mechanistic insights into how CHM may modulate bacterial-immune interactions and inflammatory pathways. This adds a new layer of understanding to the existing evidence, highlighting the potential of integrated therapies to address both local and systemic inflammation. Furthermore, the identification of core TCM treatment strategies, such as purgative methods, offers a theoretical framework for future research and clinical practice. Overall, this study fills a critical gap in the literature by providing robust evidence for the efficacy of CHM-WM combination therapy in SAP-ALI/ARDS, while also offering new perspectives on its underlying mechanisms.

Methodological Improvements: Future RCTs should prioritize concealed allocation, blinding, and standardized endpoint measures, including quality-of-life assessments. Clinical Validation: Larger sample sizes are needed to confirm the effects on mortality outcomes, and further optimization of herbal formula dosages and treatment durations is required. Mechanistic Exploration: Research should focus on the gut-lung axis, particularly bacterial-immune interactions and inflammatory mediator regulation, to deepen the understanding of TCM’s “lung-intestine interrelatedness” theory. Addressing these gaps will enhance the understanding of the mechanisms underlying the efficacy of integrated Chinese and WM in treating SAP-ALI/ARDS, fostering the integration of evidence-based medicine and translational research.

## Conclusion

Integrated CHM-WM therapy significantly improves clinical outcomes for patients with SAP-associated ALI/ARDS, particularly mitigating inflammation and accelerating disease resolution. Future research should prioritize the optimization of RCTs designs, including the implementation of blinding protocols, and investigate the molecular mechanisms underlying the gut-lung axis to further substantiate the evidence base.

## Data Availability

The original contributions presented in the study are included in the article/Supplementary Material, further inquiries can be directed to the corresponding author.

## Supplementary material

SUPPLEMENTARY FIGURE 1 Funnel plot of comparison of oxygenation index.

SUPPLEMENTARY FIGURE 2 Frequency of use of Chinese medicine included in the study.
