# Supplementary material for: Assessment of the efficacy of Chinese herbal medicine combined with western medicine for treating severe acute pancreatitis-related acute lung injury/acute respiratory distress syndrome: a systematic review and meta-analysis based on randomized controlled trials
Source: Front Pharmacol. 2025 Oct 30;16:1551652. doi: 10.3389/fphar.2025.1551652 (PMC12611793; doi:10.3389/fphar.2025.1551652)
Supplement: Supplementary file 1 [file Table1.docx]

Supplementary Table 1 Search strategy in PubMed, Embase, Cochrane Library, ProQuest, OVID, Scopus, CBM, CNKI, Duxiu, WanFang, VIP, and Web of Science.

| Database | Search strategy |
| --- | --- |
| PubMed | 1. ("Pancreatitis"[Mesh] OR ("Pancreatitis, Acute"[Title/Abstract] OR "Acute Pancreatitis"[Title/Abstract] OR "Acute Pancreatitides"[Title/Abstract] OR "Pancreatitides, Acute"[Title/Abstract] OR "Pancreatitis, Acute Edematous"[Title/Abstract] OR "Acute Edematous Pancreatitides"[Title/Abstract] OR "Edematous Pancreatitides, Acute"[Title/Abstract] OR "Edematous Pancreatitis, Acute"[Title/Abstract] OR "Pancreatitides, Acute Edematous"[Title/Abstract] OR "Acute Edematous Pancreatitis"[Title/Abstract] OR "AP"[Title/Abstract] OR "SAP"[Title/Abstract])) 2. ("Acute Lung Injury"[Mesh] OR ("Acute Lung Injuries"[Title/Abstract] OR "Lung Injuries, Acute"[Title/Abstract] OR "Lung Injury, Acute"[Title/Abstract] OR "acute respiratory distress syndrome"[Title/Abstract] OR "ALI"[Title/Abstract] OR "ARDS"[Title/Abstract])) 3. ("Medicine, Chinese Traditional"[Mesh] OR ("Zhong Yi Xue"[Title/Abstract] OR "Chung I Hsueh"[Title/Abstract] OR "Hsueh, Chung I"[Title/Abstract] OR "Traditional Medicine, Chinese"[Title/Abstract] OR "Chinese Traditional Medicine"[Title/Abstract] OR "Traditional Chinese Medicine"[Title/Abstract] OR "Chinese Medicine, Traditional"[Title/Abstract] OR "TCM"[Title/Abstract])) 4. #1 AND #2 AND #3 5. ("Randomized Controlled Trials as Topic"[Mesh] OR ("Clinical Trials, Randomized"[Title/Abstract] OR "Trials, Randomized Clinical"[Title/Abstract] OR "Controlled Clinical Trials, Randomized"[Title/Abstract] OR "RCT"[Title/Abstract] OR "randomized controlled trial"[Publication Type] OR "randomized"[Title/Abstract] OR "placebo"[Title/Abstract])) 6. #4 AND #5 |
| CNKI | (Topic:"Acute Pancreatitis") OR (Topic:"Severe Acute Pancreatitis") OR (Topic:"AP") OR (Topic:"SAP") AND (Topic:"Acute Lung Injury") OR (Topic:"ARDS") OR (Topic:"ALI") OR (Topic:"Acute Respiratory Distress Syndrome") AND (Topic:"Traditional Chinese Medicine") OR (Topic:"Integrative Medicine") OR (Topic:"Chinese Herbal Medicine") AND (Abstract:"Randomized Controlled Trial" OR Abstract:"RCT" OR Abstract:"Randomized") |
| CBM | 1. "Pancreatitis"[unweighted: expanded] 2. "Acute Hemorrhagic Pancreatitis"[unweighted: expanded] 3. "Pancreatitis, Acute Necrotizing"[unweighted: expanded] 4. "Acute Pancreatitis"[common fields: smart] OR "Severe Acute Pancreatitis"[common fields: smart] 5. "Acute Lung Injury"[unweighted: expanded] 6. "Acute Respiratory Distress Syndrome"[common fields: smart] 7. "ALI"[common fields: smart] OR "ARDS"[common fields: smart] 8. (#7) OR (#6) OR (#5) 9. "AP"[common fields: smart] OR "SAP"[common fields: smart] 10. (#9) OR (#4) OR (#3) OR (#2) OR (#1) 11. "Randomized Controlled Trial"[unweighted: expanded] 12. "Randomized"[common fields: smart] OR "RCT"[common fields: smart] 13. (#12) OR (#11) 14. "Traditional Chinese Medicine"[unweighted: expanded] 15. "Integrative Medicine"[unweighted: expanded] 16. "Integrative Medicine Therapy"[unweighted: expanded] 17. "Traditional Chinese Studies"[unweighted: expanded] 18. "Chinese Herbal Medicine"[unweighted: expanded] 19. "Chinese Medicinal Herbs"[unweighted: expanded] 20. (#19) OR (#18) OR (#17) OR (#16) OR (#15) OR (#14) 21. (#20) AND (#13) AND (#10) AND (#8) |
| Cochrane library | 1. MeSH descriptor: [Pancreatitis] explode all trees 2. (Pancreatitis, Acute):ti,ab,kw OR (Acute Pancreatitis):ti,ab,kw OR (Acute Pancreatitides):ti,ab,kw OR (Pancreatitides, Acute):ti,ab,kw OR (Pancreatitis, Acute Edematous):ti,ab,kw OR (Acute Edematous Pancreatitides):ti,ab,kw OR (Edematous Pancreatitides, Acute):ti,ab,kw OR (Edematous Pancreatitis, Acute):ti,ab,kw OR (Pancreatitides, Acute Edematous):ti,ab,kw OR (Acute Edematous Pancreatitis):ti,ab,kw OR (AP):ti,ab,kw OR (SAP):ti,ab,kw OR (Peripancreatic Fat Necrosis):ti,ab,kw OR (Fat Necrosis, Peripancreatic):ti,ab,kw OR (Necrosis, Peripancreatic Fat):ti,ab,kw OR (Peripancreatic Fat Necroses):ti,ab,kw OR (Pancreatic Parenchymal Edema):ti,ab,kw OR (Edema, Pancreatic Parenchymal):ti,ab,kw OR (Pancreatic Parenchymal Edemas):ti,ab,kw OR (Parenchymal Edema, Pancreatic):ti,ab,kw OR (Pancreatic Parenchyma with Edema):ti,ab,kw 3. #1 OR #2 4. MeSH descriptor: [Acute Lung Injury] explode all trees 5. (Acute Lung Injuries):ti,ab,kw OR (Lung Injuries, Acute):ti,ab,kw OR (Lung Injury, Acute):ti,ab,kw OR (acute respiratory distress syndrome):ti,ab,kw OR (ALI):ti,ab,kw OR (ARDS):ti,ab,kw 6. #4 OR #5 7. #3 AND #6 8. MeSH descriptor: [Medicine, Chinese Traditional] explode all trees 9. (Zhong Yi Xue):ti,ab,kw OR (Chung I Hsueh):ti,ab,kw OR (Hsueh, Chung I):ti,ab,kw OR (Traditional Medicine, Chinese):ti,ab,kw OR (Chinese Traditional Medicine):ti,ab,kw OR (Traditional Chinese Medicine):ti,ab,kw 10. #8 OR #9 11. #7 AND #10 |
| Duxiu | (T= "Acute Pancreatitis" \| S= "Acute Pancreatitis" \| T= "Severe Acute Pancreatitis" \| S= "Severe Acute Pancreatitis" \| T= "AP" \| S= "AP" \| T= "SAP" \| S= "SAP") * (T= "Acute Lung Injury" \| S= "Acute Lung Injury" \| T= "Acute Respiratory Distress Syndrome" \| S= "Acute Respiratory Distress Syndrome" \| T= "ALI" \| S= "ALI" \| T= "ARDS" \| S= "ARDS") * (T= "Traditional Chinese Medicine" \| S= "Traditional Chinese Medicine" \| T= "Chinese Herbal Medicine" \| S= "Chinese Herbal Medicine" \| T= "Integrative Medicine" \| S= "Integrative Medicine") * (T= "Randomized Controlled Trial" \| S= "Randomized Controlled Trial" \| T= "RCT" \| S= "RCT" \| T= "Randomized" \| S= "Randomized") |
| OVID | 1. (Pancreatitis OR "Pancreatitis, Acute" OR "Acute Pancreatitis" OR "Acute Pancreatitides" OR "Pancreatitides, Acute" OR "Pancreatitis, Acute Edematous" OR "Acute Edematous Pancreatitides" OR "Edematous Pancreatitides, Acute" OR "Edematous Pancreatitis, Acute" OR "Pancreatitides, Acute Edematous" OR "Acute Edematous Pancreatitis" OR "AP" OR "SAP" OR "Peripancreatic Fat Necrosis" OR "Fat Necrosis, Peripancreatic" OR "Necrosis, Peripancreatic Fat" OR "Peripancreatic Fat Necroses" OR "Pancreatic Parenchymal Edema" OR "Edema, Pancreatic Parenchymal" OR "Pancreatic Parenchymal Edemas" OR "Parenchymal Edema, Pancreatic" OR "Pancreatic Parenchyma with Edema").ti,ab,kw 2. ("Acute Lung Injuries" OR "Acute Lung Injury" OR "Lung Injuries, Acute" OR "Lung Injury, Acute" OR "acute respiratory distress syndrome" OR "ALI" OR "ARDS").ti,ab,kw 3. ("Medicine, Chinese Traditional" OR "Zhong Yi Xue" OR "Chung I Hsueh" OR "Hsueh, Chung I" OR "Traditional Medicine, Chinese" OR "Traditional Chinese Medicine" OR "Chinese Traditional Medicine" OR "Chinese Medicine, Traditional" OR "TCM").ti,ab,kw 4. #1 AND #2 5. ("randomized controlled trial" OR "Clinical Trials, Randomized" OR "Trials, Randomized Clinical" OR "Controlled Clinical Trials, Randomized" OR "RCT" OR "Randomized" OR "placebo").ti,ab,kw 6. #3 AND #4 AND #5 |
| Embase | 1. 'pancreatitis'/exp OR pancreatitis 2. ('pancreatitis, acute':ab,ti OR 'acute pancreatitis':ab,ti OR 'acute pancreatitides':ab,ti OR 'pancreatitides, acute':ab,ti OR 'pancreatitis, acute edematous':ab,ti OR 'acute edematous pancreatitides':ab,ti OR 'edematous pancreatitides, acute':ab,ti OR 'edematous pancreatitis, acute':ab,ti OR 'pancreatitides, acute edematous':ab,ti OR 'acute edematous pancreatitis':ab,ti OR 'ap':ab,ti OR 'sap':ab,ti OR 'peripancreatic fat necrosis':ab,ti OR 'fat necrosis, peripancreatic':ab,ti OR 'necrosis, peripancreatic fat':ab,ti OR 'peripancreatic fat necroses':ab,ti OR 'pancreatic parenchymal edema':ab,ti OR 'edema, pancreatic parenchymal':ab,ti OR 'pancreatic parenchymal edemas':ab,ti OR 'parenchymal edema, pancreatic':ab,ti OR 'pancreatic parenchyma with edema':ab,ti) 3. #1 OR #2 4. acute AND lung AND injury 5. ('acute lung injuries':ab,ti OR 'lung injuries, acute':ab,ti OR 'lung injury, acute':ab,ti OR 'acute respiratory distress syndrome':ab,ti OR 'ali':ab,ti OR 'ards':ab,ti) 6. #4 OR #5 7. #3 AND #6 8. 'chinese medicine' 9. ('zhong yi xue':ab,ti OR 'chung i hsueh':ab,ti OR 'hsueh, chung i':ab,ti OR 'traditional medicine, chinese':ab,ti OR 'chinese traditional medicine':ab,ti OR 'traditional chinese medicine':ab,ti OR 'tcm':ab,ti OR 'chinese medicine, traditional':ab,ti) 10. #8 OR #9 11. #7 AND #10 12. 'clinical trials, randomized':ab,ti OR 'trials, randomized clinical':ab,ti OR 'controlled clinical trials, randomized':ab,ti OR 'rct':ab,ti OR 'randomized controlled trial':ab,ti OR 'randomized':ab,ti OR 'placebo':ab,ti 13. #11 AND #12 |
| Web of Science | 1. Topic: ("Acute lung injury") 2. Topic: ("Acute lung injury" OR "Acute Lung Injuries" OR "Lung Injury, Acute" OR "acute respiratory distress syndrome" OR "ALI" OR "ARDS" OR "Lung Injuries, Acute") 3. Topic: ("Pancreatitis" OR "Pancreatitis, Acute" OR "Acute Pancreatitis" OR "Acute Pancreatitides" OR "Pancreatitides, Acute" OR "Pancreatitis, Acute Edematous" OR "Acute Edematous Pancreatitides" OR "Edematous Pancreatitides, Acute" OR "Edematous Pancreatitis, Acute" OR "Pancreatitides, Acute Edematous" OR "Acute Edematous Pancreatitis" OR "AP" OR "SAP" OR "Peripancreatic Fat Necrosis" OR "Fat Necrosis, Peripancreatic" OR "Necrosis, Peripancreatic Fat" OR "Peripancreatic Fat Necroses" OR "Pancreatic Parenchymal Edema" OR "Edema, Pancreatic Parenchymal" OR "Pancreatic Parenchymal Edemas" OR "Parenchymal Edema, Pancreatic" OR "Pancreatic Parenchyma with Edema") 4. #2 AND #3 5. Topic: ("Medicine, Chinese Traditional" OR "Zhong Yi Xue" OR "Chung I Hsueh" OR "Hsueh, Chung I" OR "Traditional Medicine, Chinese" OR "Chinese Traditional Medicine" OR "Traditional Chinese Medicine" OR "Chinese Medicine, Traditional") 6. Topic: ("Clinical Trials, Randomized" OR "Trials, Randomized Clinical" OR "Controlled Clinical Trials, Randomized" OR "RCT" OR "randomized controlled trial" OR "randomized" OR "placebo") 7. #4 AND #5 AND #6 |
| ProQuest | S1 AB,TI("Pancreatitis" OR "Pancreatitis, Acute" OR "Acute Pancreatitis" OR "Acute Pancreatitides" OR "Pancreatitides, Acute" OR "Pancreatitis, Acute Edematous" OR "Acute Edematous Pancreatitides" OR "Edematous Pancreatitides, Acute" OR "Edematous Pancreatitis, Acute" OR "Pancreatitides, Acute Edematous" OR "Acute Edematous Pancreatitis" OR "AP" OR "SAP" OR "Peripancreatic Fat Necrosis" OR "Fat Necrosis, Peripancreatic" OR "Necrosis, Peripancreatic Fat" OR "Peripancreatic Fat Necroses" OR "Pancreatic Parenchymal Edema" OR "Edema, Pancreatic Parenchymal" OR "Pancreatic Parenchymal Edemas" OR "Parenchymal Edema, Pancreatic" OR "Pancreatic Parenchyma with Edema")  S2 AB,TI("Acute lung injury" OR "Acute Lung Injuries" OR "Lung Injuries, Acute" OR "Lung Injury, Acute" OR "ALI" OR "acute respiratory distress syndrome" OR "ARDS")  S3 [S1] AND [S2]  S4 AB,TI("Medicine, Chinese Traditional" OR "Zhong Yi Xue" OR "Chung I Hsueh" OR "Hsueh, Chung I" OR "Traditional Medicine, Chinese" OR "Chinese Traditional Medicine" OR "Traditional Chinese Medicine" OR "Chinese Medicine, Traditional" OR "TCM")  S5 AB,TI("Clinical Trials, Randomized" OR "randomized controlled trial" OR "Trials, Randomized Clinical" OR "Controlled Clinical Trials, Randomized" OR "Randomized" OR "RCT" OR "placebo")  S6 [S3] AND [S4] AND [S5] |
| Scopus | 1. TITLE-ABS-KEY("Pancreatitis" OR "Pancreatitis, Acute" OR "Acute Pancreatitis" OR "Acute Pancreatitides" OR "Pancreatitides, Acute" OR "Pancreatitis, Acute Edematous" OR "Acute Edematous Pancreatitides" OR "Edematous Pancreatitides, Acute" OR "Edematous Pancreatitis, Acute" OR "Pancreatitides, Acute Edematous" OR "Acute Edematous Pancreatitis" OR "AP" OR "SAP" OR "Peripancreatic Fat Necrosis" OR "Fat Necrosis, Peripancreatic" OR "Necrosis, Peripancreatic Fat" OR "Peripancreatic Fat Necroses" OR "Pancreatic Parenchymal Edema" OR "Edema, Pancreatic Parenchymal" OR "Pancreatic Parenchymal Edemas" OR "Parenchymal Edema, Pancreatic" OR "Pancreatic Parenchyma with Edema") 2. TITLE-ABS-KEY("Acute Lung Injury" OR "Acute Lung Injuries" OR "Lung Injuries, Acute" OR "Lung Injury, Acute" OR "acute respiratory distress syndrome" OR "ALI" OR "ARDS") 3. TITLE-ABS-KEY("Medicine, Chinese Traditional" OR "Zhong Yi Xue" OR "Chung I Hsueh" OR "Hsueh, Chung I" OR "Traditional Medicine, Chinese" OR "Chinese Traditional Medicine" OR "Traditional Chinese Medicine" OR "Chinese Medicine, Traditional" OR "TCM") 4. #1 AND #2 5. TITLE-ABS-KEY("Randomized Controlled Trials" OR "Clinical Trials, Randomized" OR "Trials, Randomized Clinical" OR "Controlled Clinical Trials, Randomized" OR "RCT" OR "randomized controlled trial" OR "randomized" OR "placebo") 6. #3 AND #4 AND #5 |
| VIP | ((((((((((((Title or Keywords = "Acute Pancreatitis" OR Title or Keywords = "acute pancreatitis") OR Title or Keywords = "AP" OR Title or Keywords = "SAP") AND (((((((Title or Keywords = "Acute Lung Injury" OR Title or Keywords = "acute lung injury") OR Title or Keywords = "acute lung injury" OR Title or Keywords = "急性肺损伤" OR Title or Keywords = "acute respiratory distress syndrome" OR Title or Keywords = "ALI" OR Title or Keywords = "ARDS"))) AND ((Abstract = "RCT" OR Abstract = "Randomized Controlled Trial" OR Abstract = "randomized"))) AND (((Title or Keywords = "Traditional Chinese Medicine" OR Title or Keywords = "Traditional Chinese Medicine" OR Title or Keywords = "Chinese Herbal Medicine") OR Title or Keywords = "Integrative Medicine"))) |
| Wanfang | Topic: ("Acute Pancreatitis" OR "Severe Acute Pancreatitis" OR "AP" OR "SAP") AND Topic: ("Acute Lung Injury" OR "Acute Respiratory Distress Syndrome" OR "ALI" OR "ARDS") AND Topic: ("Traditional Chinese Medicine" OR "Chinese Herbal Medicine" OR "Integrative Medicine") AND Topic: ("Randomized Controlled Trial" OR "Randomized" OR "RCT") |

CBM, Chinese Biomedical Literature Database SinoMed; CNKI, China National Knowledge Network journal full-text database; Wan Fang, Wan fang data knowledge service platform; VIP, VIP Chinese science and technology periodical database.
