# Supplementary figures and images for: Assessment of the efficacy of Chinese herbal medicine combined with western medicine for treating severe acute pancreatitis-related acute lung injury/acute respiratory distress syndrome: a systematic review and meta-analysis based on randomized controlled trials
Source: Front Pharmacol. 2025 Oct 30;16:1551652. doi: 10.3389/fphar.2025.1551652 (PMC12611793; doi:10.3389/fphar.2025.1551652)

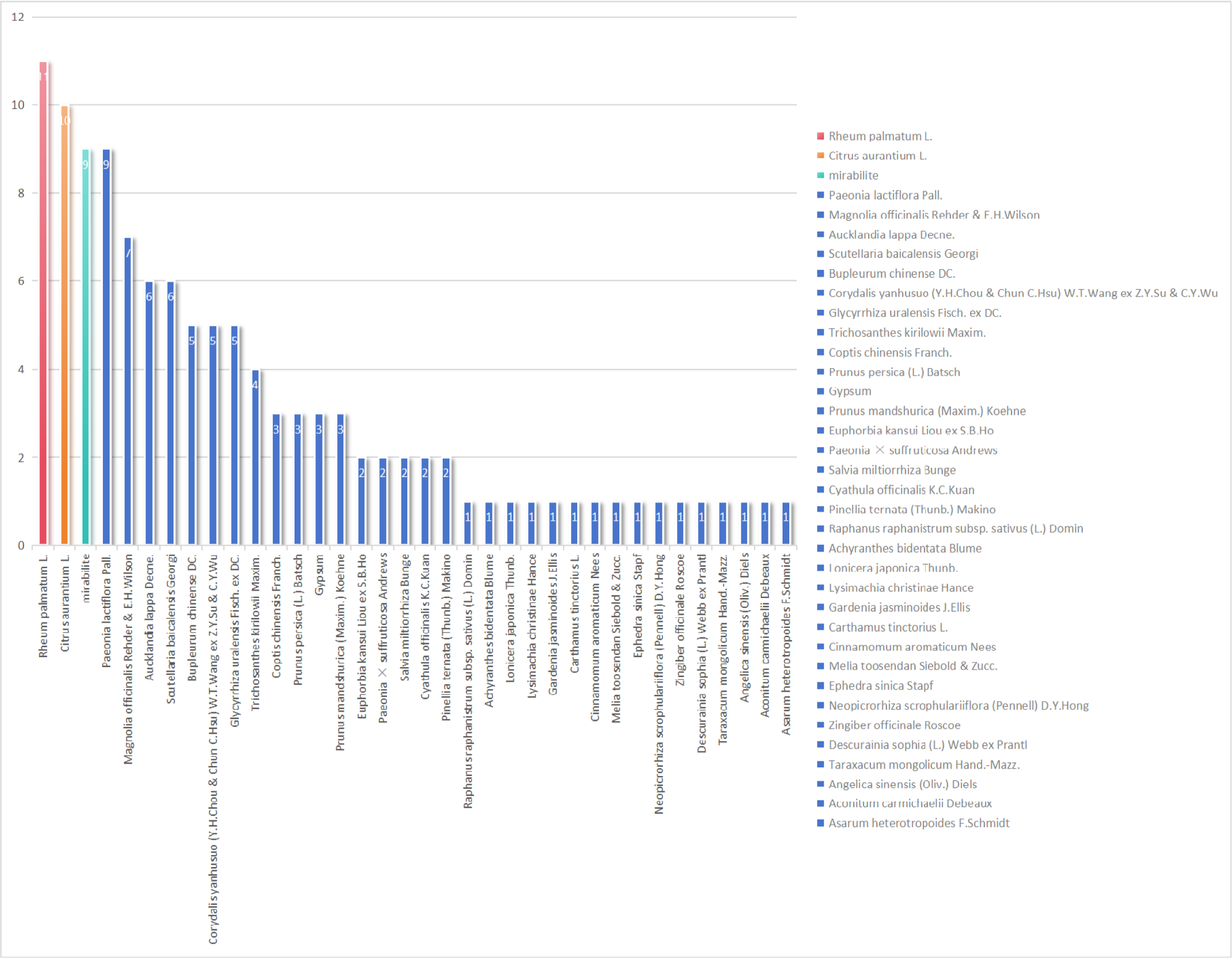

Supplement: Supplementary file 2 [file Image2.tif]

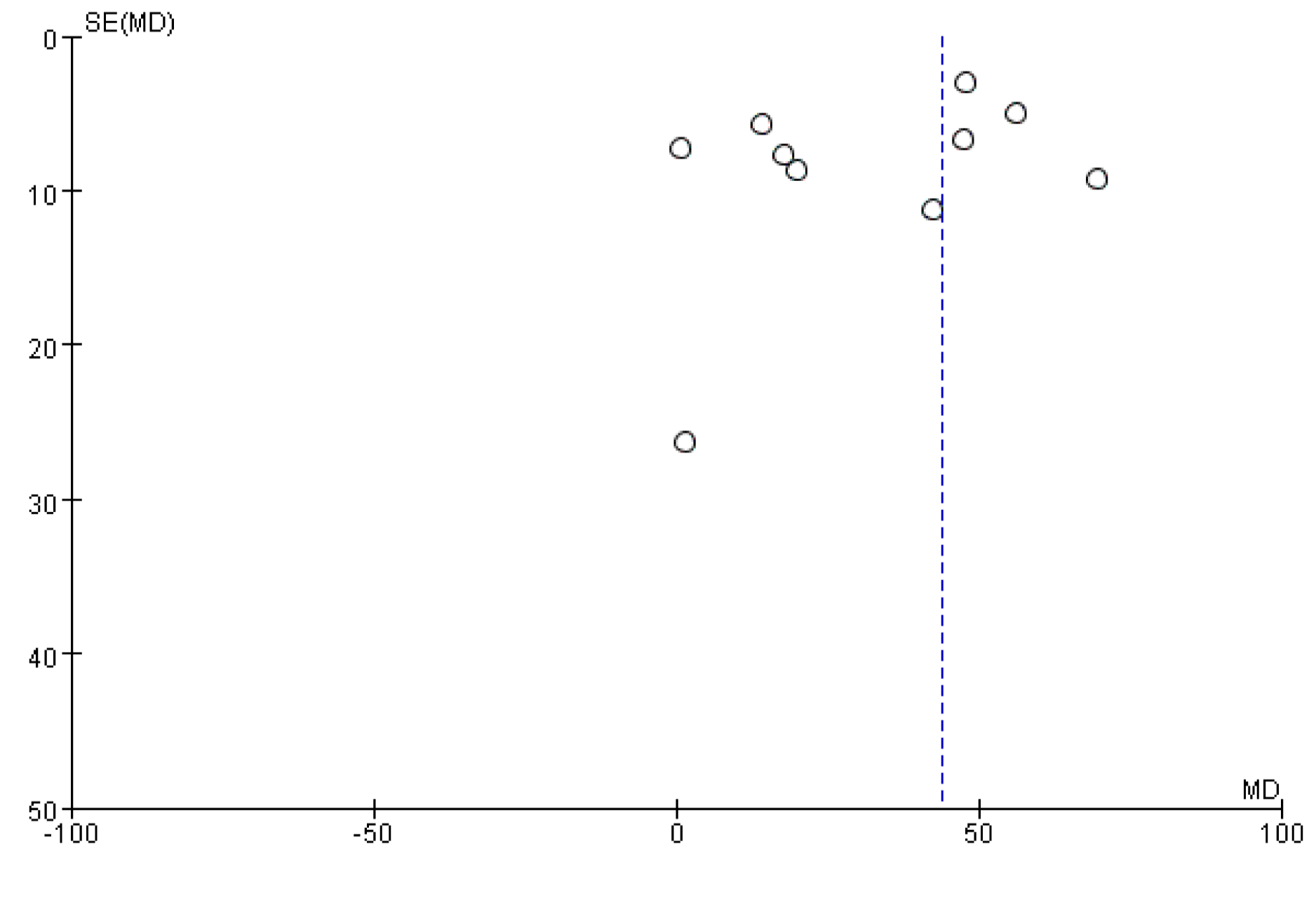

Supplement: Supplementary file 3 [file Image1.tif]
